# De Novo Engineering of Pd-Metalloproteins and Their Use as Intracellular Catalysts

**DOI:** 10.1021/jacsau.4c00379

**Published:** 2024-06-23

**Authors:** Soraya Learte-Aymamí, Laura Martínez-Castro, Carmen González-González, Miriam Condeminas, Pau Martin-Malpartida, María Tomás-Gamasa, Sandra Baúlde, José R. Couceiro, Jean-Didier Maréchal, Maria J. Macias, José L. Mascareñas, M. Eugenio Vázquez

**Affiliations:** † Centro Singular de Investigación en Química Biolóxica e Materiais Moleculares (CiQUS), Departamento de Química Orgánica, Universidade de Santiago de Compostela, Santiago de Compostela 15705, Spain; ‡ Insilichem, Departament de Química, Universitat Autónoma de Barcelona, Cerdanyola 08193, Spain; § Institute for Research in Biomedicine (IRB Barcelona), The Barcelona Institute of Science and Technology (BIST), Baldiri Reixac, 10, Barcelona 08028, Spain; ∥ Academic institutional affiliation:Department of Medicine and Life Sciences, Universitat Pompeu Fabra (MELIS-UPF), Carrer del Doctor Aiguader 88, Barcelona 08003, Spain; ⊥ Institució Catalana de Recerca i Estudis Avançats (ICREA), Passeig Lluís Companys 23, Barcelona 08010, Spain

**Keywords:** intracellular catalysis, peptide engineering, metallopeptides, grafting, palladium, organometallic catalysis

## Abstract

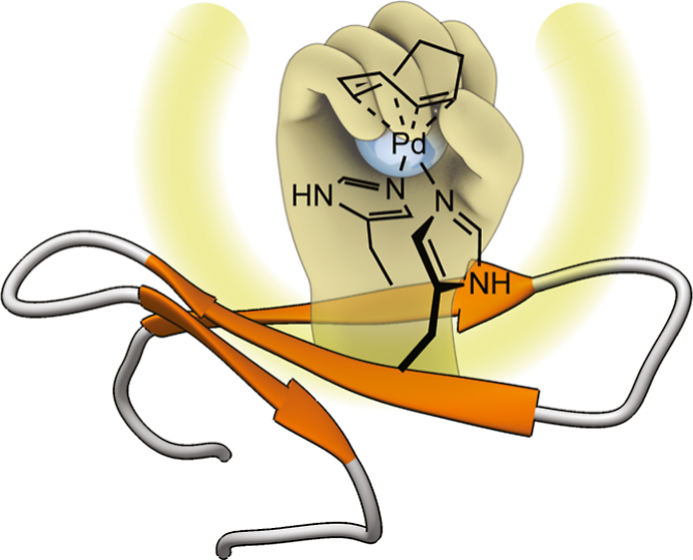

The development of transition metal-based catalytic platforms that promote bioorthogonal reactions inside living cells remains a major challenge in chemical biology. This is particularly true for palladium-based catalysts, which are very powerful in organic synthesis but perform poorly in the cellular environment, mainly due to their rapid deactivation. We now demonstrate that grafting Pd(II) complexes into engineered β-sheets of a model WW domain results in cell-compatible palladominiproteins that effectively catalyze depropargylation reactions inside HeLa cells. The concave shape of the WW domain β-sheet proved particularly suitable for accommodating the metal center and protecting it from rapid deactivation in the cellular environment. A thorough NMR and computational study confirmed the formation of the metal-stapled peptides and allowed us to propose a three-dimensional structure for this novel metalloprotein motif.

## Introduction

**Figure 1:**
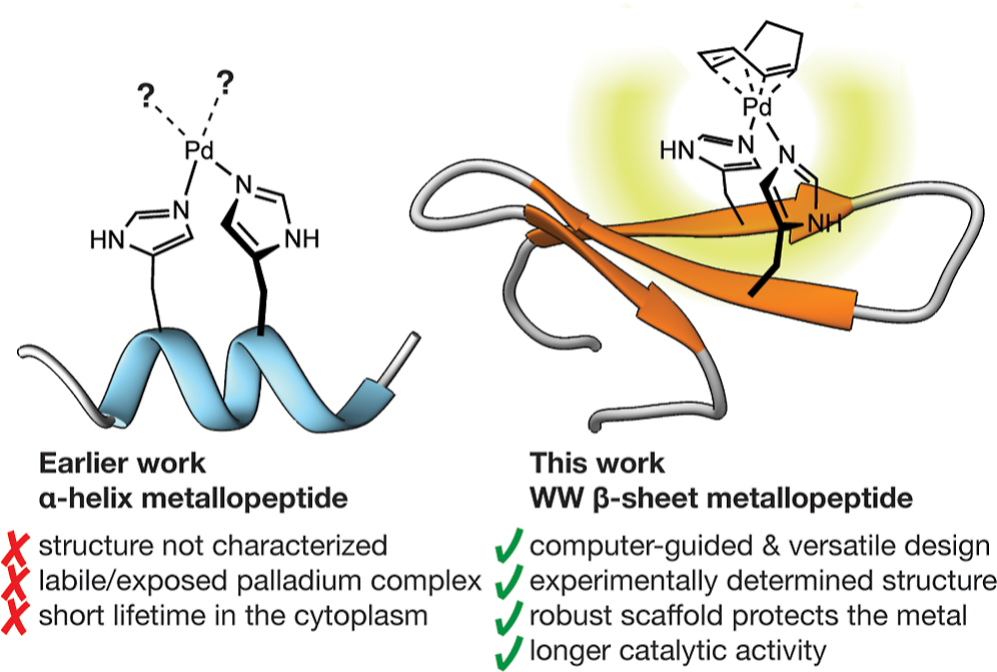
Palladopeptide frameworks with grafted bis-histidine residues. The concave β-sheet structure provides greater protection to the catalytic metal center and its extended surface offers many alternatives to engineer pairs of chelating residues.

Bioorthogonal transformations are powerful tools for the study and modification of biological systems.^1−5^ Besides the now classic ligation reactions involving high-energy (strained) reactants, there is a growing interest in the implementation of reactions facilitated by transition metal complexes in cellular settings.^6−10^ In this context, palladium-based catalysts are particularly attractive due to their leading role in synthetic organic chemistry.^11,12^ However, these complexes tend to be rapidly deactivated in the intracellular environment.^13−15^ For example, [PdCl_2_(COD)] effectively catalyzes depropargylation reactions in aqueous media in vitro, but it fails to promote this transformation in cells.^16−18^ We envisioned that integrating the metal center into a peptide scaffold might extend the lifetime of the active catalyst and facilitate the cellular internalization. Moreover, peptides are inherently biocompatible, they can be easily synthesized using solid phase methodologies and are modular, so they can be easily tweaked to fine-tune their structure and properties and engineer metal coordination sites.^19−22^ We have recently shown that grafting a pair of His residues in consecutive helical turns of a synthetic α-helical peptide creates a suitable coordination site for Pd(II), and we found that it can promote depropargylation reactions in living cells (Figure 1a).^23^ Unfortunately, the experiment fails when the cells are incubated with the metallopeptide for 1 h before adding the substrate, which suggests the deactivation of the catalysts in the intracellular media, likely due to the lability of the peptide coordination and/or the solvent exposure of the metal center. Despite these limitations, the success of the metal-stapling strategy encouraged us to explore alternative platforms to develop more robust and long-lasting palladium–peptide complexes that can function as cell-compatible catalysts. In this context, we envisioned that multistrand β-sheet structures might offer more opportunities to engineer robust catalysts, due to their concave structure, which can cradle the catalytic center, and their extended surface area, which offers many alternatives to strategically position chelating residues (Figure 1b).

Here, we demonstrate the feasibility of this approach by reporting a synthetic palladoprotein based on the triple-stranded β-sheet of a WW domain that can efficiently catalyze depropargylation reactions in HeLa cells. This catalytic platform, which we have structurally characterized by NMR and computational data, represents a novel type of metallopeptide motif and can be considered as an *in cellulo* active proto-metalloenzyme.

## Results and Discussion

### Design of the β-Sheet Platform

The WW domain is a small protein with a triple-stranded antiparallel β-sheet that acts as a signaling domain by binding to polyproline-rich sequences.^24^ Because of its small size (<40 residues), compact fold, and stability,^25^ the WW domain is a good model for protein engineering^26−28^ and an attractive platform to test our metal-grafting strategy to generate catalytic metallopeptides.^29^ The β-sheet scaffold provides ample opportunities to engineer pairs of metal-chelating His residues, and its concave shape might provide greater protection to the catalytic center.

**Figure 2:**
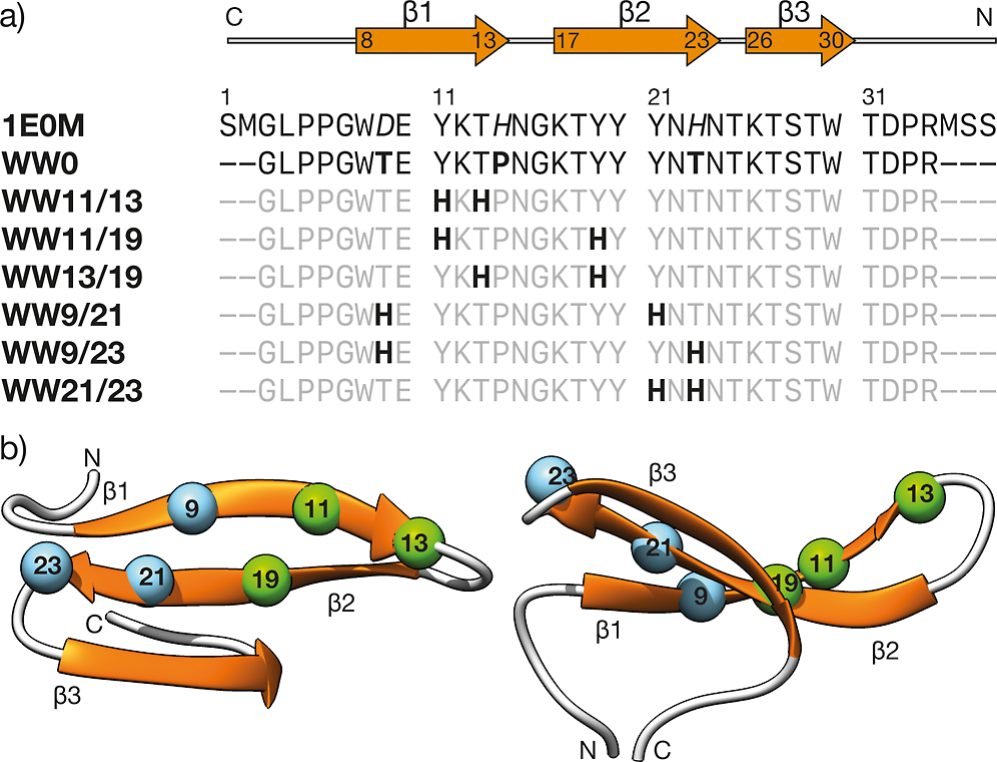
(a) Sequences of the WW reference 1E0M and the designed WW peptides. The metal-coordinating His residues are in bold and the rest of the sequence in gray for clarity. Peptides are named according to the positions of the grafted His residues; (b) top and side view of the reference 1E0M WW domain showing the concave structure of the β-sheet, and indicating the positions 9, 11, 13, 19, 21, and 23, where the His residues are grafted. The two sets of residues that define different metal binding sites are highlighted in light blue and green. Residue numbering according to the reference PDB structure 1E0M.

As a starting point for our designs, we chose the WW prototype described by Macias et al. (PDB entry 1E0M).^30^ To avoid any interference with the coordination of the metal ion, we replaced residues His14 and His23 in the 1E0M sequence with Pro and Thr, respectively, which are residues commonly found at these positions in natural WW domains. In addition, preliminary tests showed us that the residue Asp9 in the 1E0M sequence has a high tendency to form aspartimides during solid-phase peptide synthesis,^31^ so we replaced it with a Thr, which is also typically found at this position in other native WW domains. Finally, the sequence 1E0M was truncated by removing the two N-terminal residues (Ser-Met) and three C-terminal residues (Met-Ser-Ser), as it has been shown that synthetic WW domains lacking these residues have been shown to retain the natural WW fold. These modifications resulted in our reference sequence **WW0** (Figure 2). Next, we combined visual inspection with computational prediction and 3D modeling to identify the best positions for grafting the pairs of His residues in the **WW0** β-sheet to create Pd(His)_2_ sites.^32^ More specifically, we screened the **WW0** scaffold with our recently released metal-binding site predictor *BioMetAll* (see page S17 of the Supporting Information for details).^33^ From this analysis, we concluded that the best candidates for grafting the pair of His residues in the concave face of the β-sheet were the adjacent positions *i*, *i* + 3 in the same strand, namely **WW11/13** and **WW21/23**; directly facing each other, as in **WW9/21** and **WW11/19**; or diagonally placed across adjacent strands, **WW9/23** and **WW13/19**. These *de novo* sequences can be grouped into two families that define distinct metal binding sites in the β1-β2 turn (Figure 2b, positions 11, 13, and 19, highlighted in green), and at the opposite end of the β1-β2 hairpin—next to the β2-β3 turn—(Figure 2b, positions 9, 21, and 23, shown in light blue).

### Pd(II) Binding to the His-Grafted WW Peptides

The designed peptides were assembled following standard Fmoc/*t*Bu solid-phase MW-assisted peptide synthesis protocols,^34−36^ purified by reverse-phase HPLC, and their identity confirmed by HPLC-MS(ESI) (Supporting Information, Figure S2). With the set of WW peptides in hand, we first confirmed their suitability for metal binding by incubating each of the peptides in water with [PdCl_2_(COD)] in a 1:1 ratio for 1 h and analyzing the resulting mixtures by HPLC-MS(ESI). As expected, the analysis of the mixture of [PdCl_2_(COD)] with **WW0**, which lacks His residues for coordination, showed no new MS peaks corresponding to a coordination complex. 1D NMR of this peptide also shows no changes upon addition of the precursor metal salt. In contrast, the grafted peptides containing pairs of histidine residues showed, in all cases, new MS peaks consistent with the formation of their palladium complexes (Supporting Information, Figure S3). A control peptide **WW19**, with only one His residue at position 19, did not show new MS peaks consistent with metal coordination, supporting our hypothesis that bidentate coordination by two histidine residues is essential for the assembly of stable complexes (Supporting Information, Figure S3).

Despite the known limitations of circular dichroism (CD) spectroscopy when dealing with β structures,^37^ it provided additional support for the formation of the Pd(II) complexes and information on the effect of the mutations on the structure of the different His-grafted peptides. Thus, the CD spectrum of the reference sequence, **WW0**, showed the typical features of a well-folded WW domain, with a positive ellipticity band at 230 nm and negative band around 210 nm.^38,39^ As expected, the spectrum of this peptide did not change significantly upon addition of one equiv of [PdCl_2_(COD)]. The circular dichroism spectra of most His-grafted peptides suggest that they do not fold as canonical WW domains, even in the presence of [PdCl_2_(COD)] (Supporting Information, Figures S11 and S12). The exception being the peptide **WW13/19**, which after addition of the palladium salt yields a CD spectrum resembling that of **WW0**. Therefore, given the importance of maintaining the WW fold for the stability and protection of the metal center from the intracellular quenchers, we chose **WW13/19** to continue our structural and catalytic studies.

### Combined NMR and Computational Analysis of WW0, WW13/19, and Its Palladium Complex WW13/19[Pd(II)]

**Figure 3:**
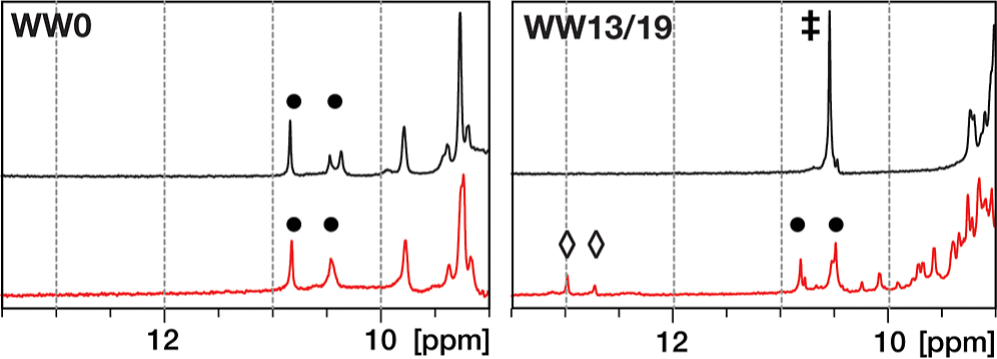
Left, detail of the ^1^H NMR spectra of the peptide **WW0** (black) and the same peptide after the addition of [PdCl_2_(COD)] (red). Right, **WW13/19** His double mutant. The peptide is mostly unfolded (black) with only one resonance for the Trp indole group (double dagger). Upon the addition of [PdCl_2_(COD)] (red), the peptide **WW13/19** folds as a canonical WW domain, as demonstrated by the presence of two Trp resonances (black circles). The palladoprotein complex shows new imidazole resonances at ∼13 ppm corresponding to the Hε of the His residues (labeled with diamonds).

To obtain more structural information on the secondary structure of the synthetic palladoproteins, the peptides **WW0** and **WW13/19** were studied by 1D NMR at room temperature in the absence and in the presence of [PdCl_2_(COD)]. As expected, peptide **WW0** showed good peak dispersion, in the absence of the palladium salt, indicating a well-folded WW domain (Supporting Information, Figure S13). The Hε peaks of both Trp residues appeared well-defined, and there were no changes in the peptide spectrum upon the addition of five equivalents of [PdCl_2_(COD)], as expected for a peptide lacking the coordinating histidines (Figure 3, left, red trace). The only difference between the two spectra is the presence of a peak at 5.8 ppm, corresponding to the protons in the double bond of the COD unit of the added [PdCl_2_(COD)] (Supporting Information, Figure S13).

In the case of the bis-histidine peptide **WW13/19**, the 1D ^1^H NMR spectrum showed poor chemical shift dispersion of the signals, with only one signal for the indoles of both Trp residues, and the absence of methyl or methylene resonances below 1 ppm, suggesting that this peptide is mostly unfolded in the absence of [PdCl_2_(COD)], under the conditions of the NMR experiments (25 °C) (Figure 3, right, black trace, and Supporting Information, Figure S14). Importantly, the addition of five equivalents of [PdCl_2_(COD)] induced a clear increase in peak dispersion, suggesting that the WW domain folds upon coordination.^29,40^ The spectrum shows new characteristic distinct resonances for each Trp Hε peak, and long–range interactions between Trp8 and Pro33 (resonances at about −0.5 ppm of the Pro side chain). Interestingly, there are also new resonances from the COD ligand in the 5–6 ppm region, consistent with the formation of the palladoprotein complex because both H in the 1,5-cyclooctadiene double bond resonate differently due to the different chemical environment of the WW structure around the COD, and the excess of [PdCl_2_(COD)] is also visible as a single resonance for both H in the double bond (Supporting Information, Figure S14). A notable feature is the presence of new resonances at 12.5 and 12.8 ppm corresponding to the coordinating His residues, (Hε2 protons), which were not observed in **WW0** (Figure 3, right, red trace and Supporting Information, Figure S13). Taken together, the NMR data at room temperature confirm that the addition of [PdCl_2_(COD)] to the bis-histidine peptide **WW13/19** triggers a folding-upon-binding process to yield a well-folded palladoprotein in which the palladium atom is coordinated to the COD ligand.

**Figure 4:**
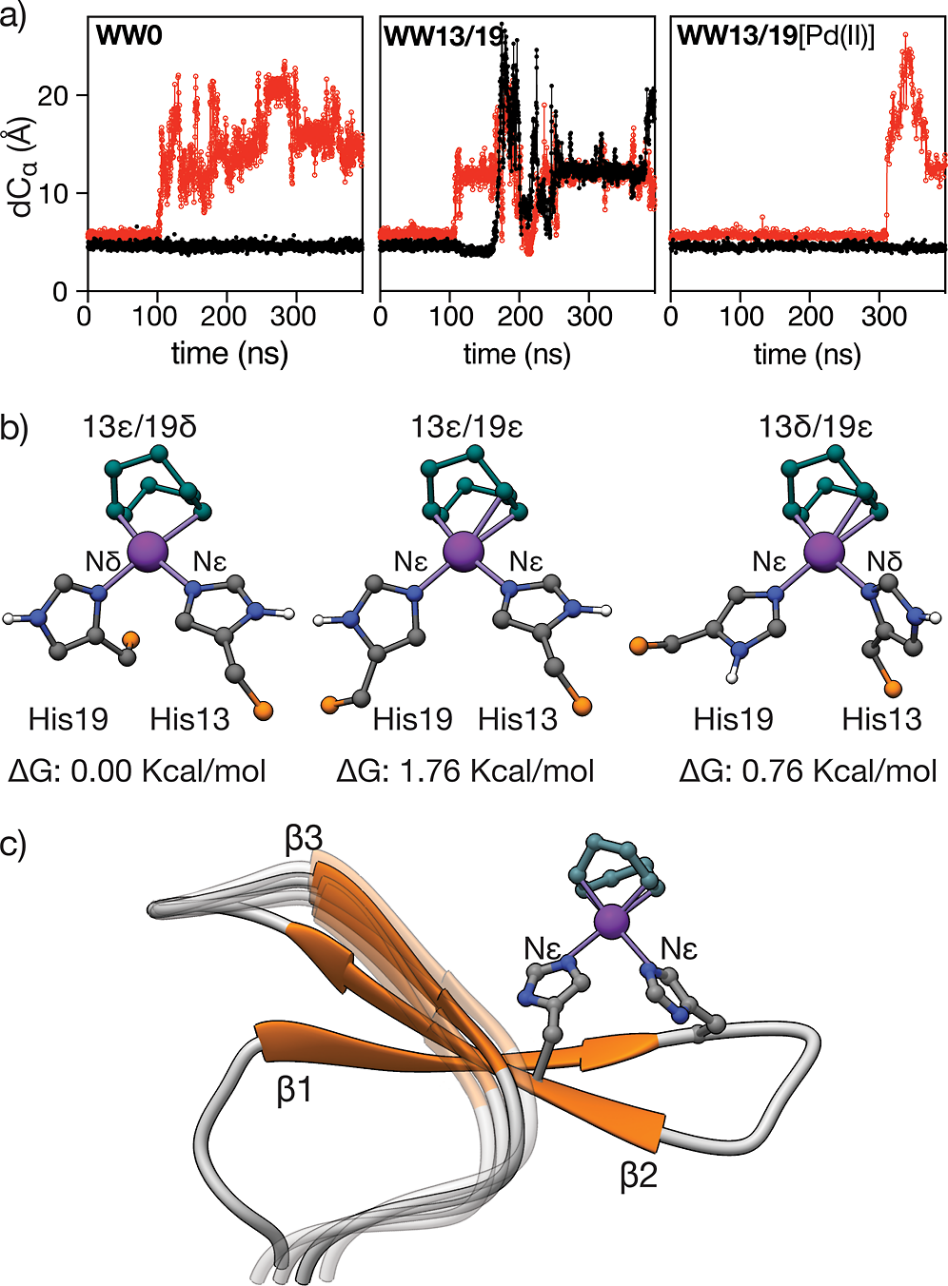
(a) Evolution of the distance between β strands along the GaMD trajectories for **WW0**, **WW13/19**, and **WW13/19**[Pd(II)]. Distances are measured between the Cα of the central residues in each strand, Glu8-Tyr18 for β1-β2 (black trace) and Tyr18-Ser26 for β2-β3 (in red). **WW13/19** shows higher flexibility and the strands fall apart rapidly, but **WW13/19**[Pd(II)] shows a highly stable β1-β2 hairpin and β3 remains stable and attached to the β-sheet for most of the simulation; (b) different coordination modes and their relative free Gibbs energy for the metal coordination resulting from DFT calculations on the model complex Pd(His)_2_COD; (c) model of **WW13/19**[Pd(II)] resulting from the combined NMR, DFT, and GaMD studies.

In agreement with the NMR experiments, extensive exploration using Gaussian Accelerated Molecular Dynamics (GaMD) under the AMBER force field 14SB,^41^ and the AMBER software, showed that the apopeptide **WW13/19** does not maintain the triple-strand configuration during most of the simulation (Figure 4a and Ramachandran plots in Supporting Information, Figure S15), whereas the palladoprotein **WW13/19**[Pd(II)] remains folded, albeit with an equilibrium between a two-stranded (β1-β2) and a three-stranded β-sheet conformation. Moreover, the broad shape of the individual Trp-Hε resonances observed in the NMR profile of the palladoprotein suggests the coexistence of His coordination tautomers. Indeed, DFT calculations performed on discrete Pd(His)_2_COD models support the formation of square-planar Pd(II) complexes in which the Pd(II) is coordinated to the Nε of one of the imidazoles and the Nδ of the other, although alternative coordination geometries cannot be ruled out due to the small energy difference between them—under 2 kcal/mol (Figure 4b and Supporting Information, page S17). With all the above information, it is possible to propose a structural model for the palladoprotein as shown in Figure 4c.

### His-Grafted Peptide WW13/19[Pd(II)] Is the Best Catalyst In Vitro

**Figure 5:**
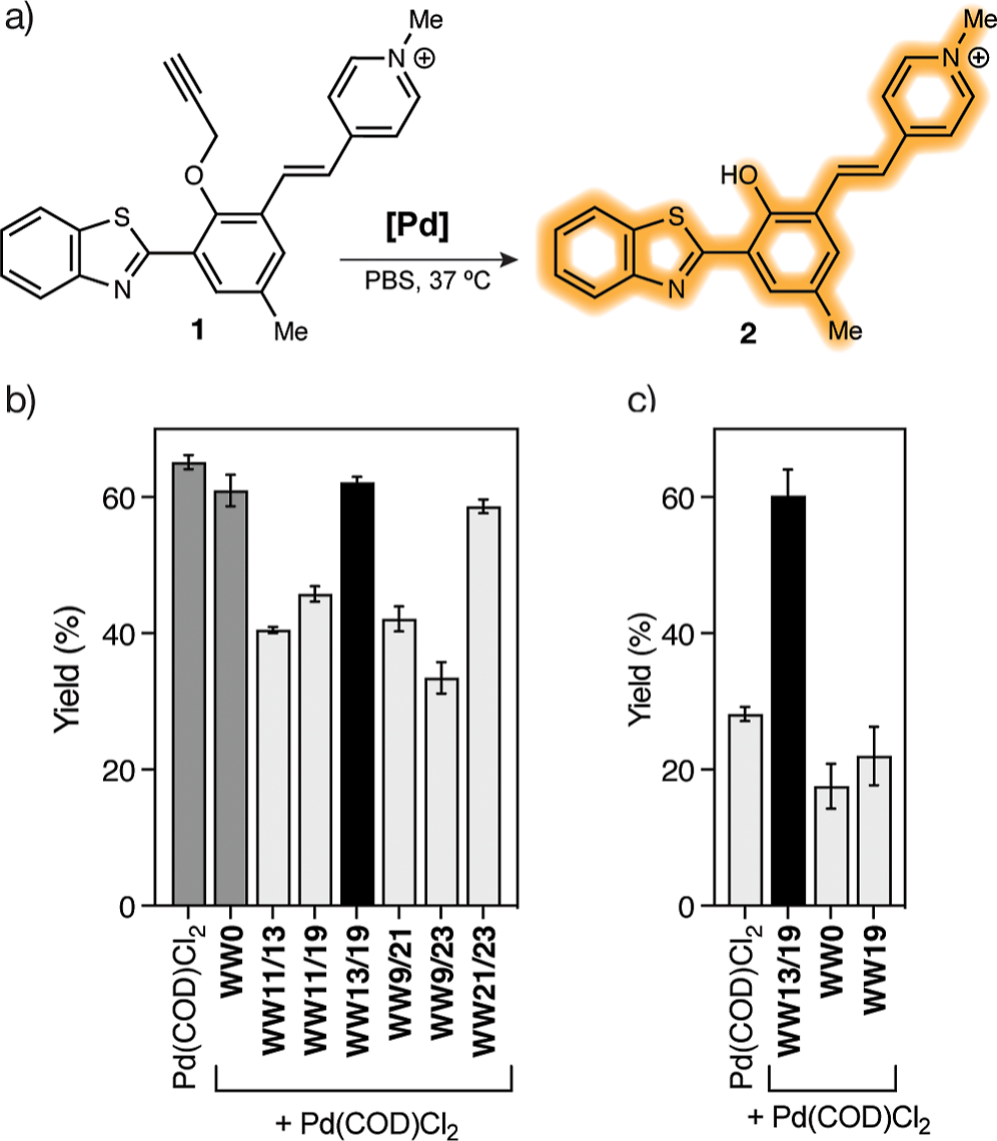
In vitro catalysis of the WW peptides. (a) Propargylated probe **1** and its deprotection reaction to yield the fluorescent product **2**; (b) mixing for 1 h a 200 μM solution of probe **1** with 20 μM mixtures of each peptide mixed with [PdCl_2_(COD)] in a 1:1 ratio for 1 h. Control experiments using [PdCl_2_(COD)] and the noncoordinating **WW0** in dark gray; (c) same experiment but with ultradiafiltration with 3 kDa Amicon centrifugal filters to remove the remaining free [PdCl_2_(COD)], before the reaction. Catalysis was performed in PBS (137 mM NaCl, 2.7 mM KCl, 8 mM Na_2_HPO_4_, 2 mM KH_2_PO_4_), and monitored by HPLC-MS(ESI).

Having confirmed that the His-grafted WW peptides form coordination complexes in the presence of [PdCl_2_(COD)], we set out to investigate whether these complexes are capable of catalyzing the depropargylation reaction of a protected model fluorogenic probe **1** (Figure 5a).^42^ To this end, we mixed 20 μM solutions of each WW peptide in Milli-Q water with [PdCl_2_(COD)] in a 1:1 ratio for 1 h. The resulting palladoprotein solutions were added to a 200 μM mixture of the fluorogenic probe **1** in PBS (137 mM NaC, 2.7 mM KCl, 8 mM Na_2_HPO_4_, 2 mM KH_2_PO_4_) and the resulting mixture was shaken at 37 °C for 24 h. HPLC-MS(ESI) analysis of the mixtures and quantification of the depropargylated product (see Supporting Information, page S19, for detailed experimental procedures), showed that the palladium complex **WW13/19**[Pd(II)] was the most active catalyst, leading to the depropargylation product in a moderately good yield of 62%, similar to that observed for the complex [PdCl_2_(COD)] in the absence of additional ligands. Pseudo-first order analysis of the reaction kinetics confirmed similar conversion rates, with *k* = 9.6 × 10^–3^ and 12.2 × 10^–3^ min^–1^ for **WW13/19**[Pd(II)] and [PdCl_2_(COD)], respectively (Supporting Information, Figure S17). Using the same protocol, but with peptide **WW0**, we also observed the depropargylation product, which was expected to have given the intrinsic catalytic activity of the unbound parent catalyst [PdCl_2_(COD)] (Figure 5b). However, when the protocol included an ultradiafiltration step with 3 kDa Amicon centrifugal filters to remove the remaining free palladium salt, the catalytic activity of the straight [PdCl_2_(COD)], and of its mixture with **WW0** decreased drastically, while the reaction using **WW13/19** is still effective. The single-His control peptide, **WW19**, also shows poor catalytic activity under these conditions, which is consistent with its inability to form a stable palladium complex (Figure 5c).

### Cellular Internalization of WW13/19 and Its Palladium Complex WW13/19[Pd(II)]

**Figure 6:**
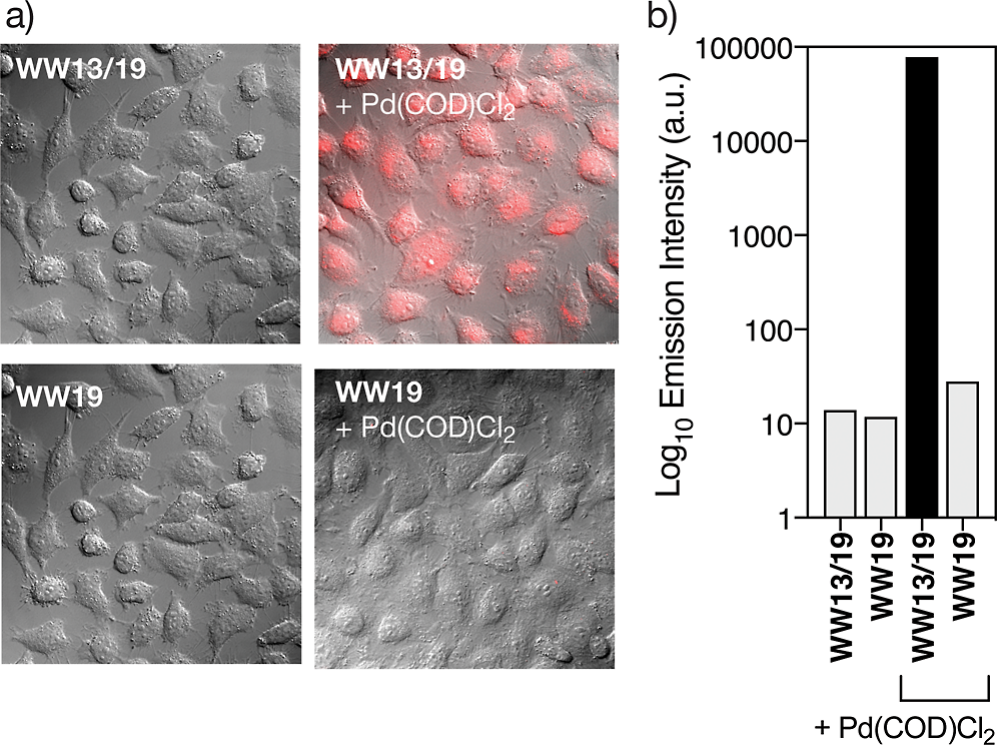
Internalization of TMR-labeled peptide **WW13/19** and its palladoprotein derivative **WW13/19**[Pd(II)]. (a) Fluorescence microscopy of HeLa cells with 5 μM solutions of the peptides **WW13/19** and **WW19** (left) and of their mixtures with [PdCl_2_(COD)] in water (right); (b) semilogarithmic plot of the intracellular TMR emission measured by cell cytometry.

As a preliminary step to study the catalytic activity of the palladoprotein **WW13/19**[Pd(II)] inside mammalian cells, we synthesized the tetramethylrhodamine-labeled peptide TMR-**WW13/19**, which would allow us to evaluate its internalization by fluorescence microscopy. Thus, we incubated HeLa cells for 1 h with 5 μM solutions of the apopeptide TMR-**WW13/19** or its Pd(II) complex, TMR-**WW13/19**[Pd(II)], which was preassembled by mixing TMR-**WW13/19** with [PdCl_2_(COD)] in water for 1 h. Interestingly, the palladoprotein TMR-**WW13/19**[Pd(II)] is better internalized than the palladium-free precursor TMR-**WW13/19**, as shown by the intensity of the intracellular fluorescence (Figure 6a).

Quantitative analysis of the internalization by cell cytometry confirmed this observation. The palladoprotein TMR-**WW13/19**[Pd(II)] internalized over 6000 times more efficiently than TMR-**WW13/19**, according to the recorded emission intensity of the TMR. The critical role of Pd(II) coordination for the internalization of these peptides is reinforced by the results with the mono-His control peptide, TMR-**WW19**, which is unable to translocate into the cellular interior, even in the presence of [PdCl_2_(COD)] (Figure 6b). Although at this time, we cannot provide a definitive explanation for the increased cellular penetration of the palladium-stapled miniprotein, it is consistent with previous observations of increased penetration as a result of conformational constraints imposed by cyclization,^43^ stapling,^44^ or coordination.^23,45^ Additional assays in the presence of various endocytosis inhibitors, suggest that the internalization of TMR-**WW13/19**[Pd(II)] is energy-dependent and most likely occurs by micropinocytosis (Supporting Information, Figure S18), accumulating inside endosomal vesicles.

### Intracellular Depropargylation Reaction Mediated by WW13/19[Pd(II)]

**Figure 7:**
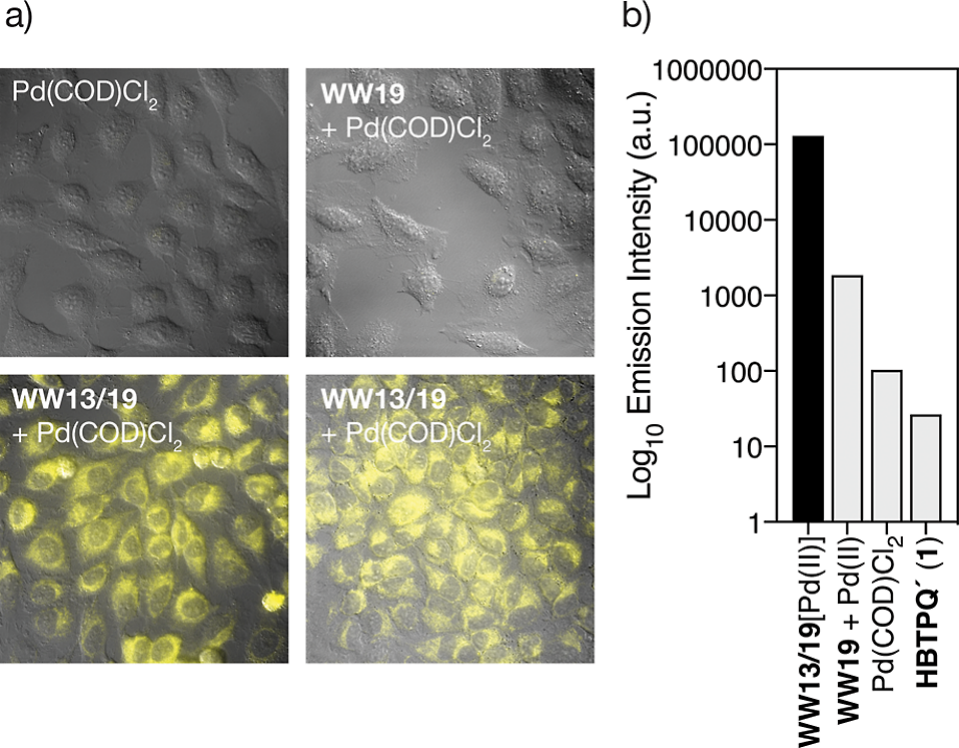
Cellular depropargylation of the fluorogenic probe **1** with **WW13/19**[Pd(II)]. (a) Fluorescence microscopy of HeLa cells incubated with a 50 μM solution of **1** for 1 h, washed twice with PBS and incubated for 1 h with [PdCl_2_(COD)], a mixture of **WW19** with [PdCl_2_(COD)], and a mixture of **WW13/19** with [PdCl_2_(COD)]. The bottom right image corresponds to the reverse experiment in which the cells are incubated first with the palladoprotein and then with the probe. Micrographs show the emission intensity at 515–700 nm upon irradiation at 385 nm; (b) quantification of the intracellular emission. Note the logarithmic scale in the intensity.

Having demonstrated the good internalization of **WW13/19**[Pd(II)], we were eager to test whether the palladoprotein could promote the depropargylation reaction inside cells. Therefore, we took advantage of the fluorogenic probe **1** tested in the in vitro experiments, which exhibits low emission in its protected form, but becomes highly fluorescent upon depropargylation, therefore allowing for easy monitoring of the catalytic deprotection reaction by fluorescence microscopy.^45^ We first incubated HeLa cells with a 50 μM solution of the probe **1**, and after 1 h we washed the cells twice with FBS-DMEM to remove any extracellular probe, and then added a 50 μM solution of freshly preformed palladoprotein **WW13/19**[Pd(II)]. After 1 h incubation, the cells showed an intense fluorescence emission under the microscope, which must be associated with the formation of the desired depropargylation product, since the protected probe is nonfluorescent under the conditions of our experiment. Importantly, control experiments using [PdCl_2_(COD)] or mixtures of [PdCl_2_(COD)] with the control peptides **WW0** and **WW19**, did not induce any intracellular emission under the same conditions (Figure 7). It is also noteworthy that the intracellular reaction also takes place with the labeled TMR-**WW13/19**[Pd(II)] (Supporting Information, Figure S19).

Encouraged by the apparent robustness of the new β-sheet-based catalyst, **WW13/19**[Pd(II)], we investigated whether it sustains catalytic cycles in the intracellular environment. We incubated HeLa cells with a 50 mM solution of the probe **1** for 1 h, washed the cells twice with PBS, added the preformed **WW13/19**[Pd(II)], and allowed the cells to stand for 1.5 h. The cells were then analyzed by ICP–MS to quantify the internalized Pd(II) and, in parallel, we performed a methanolic extraction of the cell contents,^46^ and quantified the depropargylated probe by HPLC-MS(ESI). This allowed us to calculate a turnover number of about 9 for **WW13/19**[Pd(II)], confirming the existence of catalytic cycles (Supporting Information, Figure S22).^47,48^ The intracellular catalytic depropargylation is also viable in other cell lines, such as HepG2 (see Supporting Information, Figure S20), and can also be performed with other probes, such as the challenging bis-propargyl protected form of cresyl violet, which upon depropargylation generates a product with emission in the red region of the spectrum (see Scheme S4 and Figure S21 in the Supporting Information).

As mentioned in the introduction, earlier work has shown that related α-helical palladopeptides are rather unstable and only catalyze the depropargylation reaction when the cells are incubated with the probe before adding the catalyst. Our new metalloprotein **WW13/19**[Pd(II)] is more robust and thus it catalyzes the depropargylation reaction using a reverse order of addition. Indeed, when HeLa cells were incubated with the preformed complex **WW13/19**[Pd(II)] for 1 h, washed to remove extracellular compounds, and mixed with the probe **1** for 1 h, we observed similar intracellular emission (Figure 7a, bottom right).

## Conclusions

In this work, we have described an unprecedented artificial metalloprotein that could be considered as a non-natural proto-metalloenzyme, since it promotes a catalytic depropargylation reaction in living mammalian cells, although still with modest activity. This miniprotein was thoroughly characterized by a combination of NMR and computational studies, which confirmed the key role of the palladium staple to ensure a well-folded domain. These results demonstrate the potential of compact β-sheet domains in the design of artificial catalytic metalloproteins that function in living cells and show that our strategy, halfway between conventional transition metal catalysts and artificial metalloenzymes, offers a powerful alternative for implementing organometallic catalysts in biological environments.

## Methods

### Peptide Synthesis

Peptides were synthesized according to standard Fmoc peptide synthesis protocols on H-Rink amide ChemMatrix resin using a *Liberty Lite* automatic microwave-assisted peptide synthesizer. Amino acids were coupled with 5-fold excess DIC as activator, Oxime as base, and DMF as solvent. Coupling was performed for 4 min at 90 °C. Deprotection of the temporary Fmoc protecting group was performed by treating the resin with 20% piperidine in DMF for 1 min at 75 °C. Cleavage/deprotection: resin-bound peptide was treated with 900 μL TFA, 50 μL CH_2_Cl_2_, 25 μL H_2_O, and 25 μL TIS (1 mL cocktail/40 mg resin) for 2 h. The resin was filtered, and the cleavage mixture was added to ice-cold diethyl ether. After 30 min, the precipitate was centrifuged and washed again with ice-cold ether. The solid residue was dried under argon and redissolved in water. Peptides were analyzed by analytical UHPLC-MS on an Agilent 1200 series LC/MS using a Phenomenex SB C_18_ (1.8 μm, 2.1 × 50 mm) analytical column. Standard analytical UHPLC conditions consisted of a linear gradient from 5 to 95% solvent B for 20 min at a flow rate of 0.35 mL/min (A: water with 0.1% TFA, B: acetonitrile with 0.1% TFA). Compounds were detected by UV absorption at 222, 270, and 330 nm. Electrospray ionization mass spectrometry (ESI/MS) was performed using an Agilent 6120 Quadrupole LC/MS model in positive scan mode with direct injection of the purified peptide solution into the MS detector. Peptide purification was performed on semipreparative RP-HPLC with an Agilent 1100 series LC equipped with a UV–visible detector using a Phenomenex Luna-C_18_ (250 × 10 mm) reversed-phase column. Standard conditions for purification by RP-HPLC consisted of a linear gradient of 5 to 75% B over 40 min at a flow rate of 4 mL/min (A: water with 0.1% TFA, B: acetonitrile with 0.1% TFA). Collected fractions containing pure products were freeze-dried.

### NMR of the WW Peptide and Its Pd Complex

NMR experiments were recorded on a Bruker AVANCE III 600 MHz spectrometer (IRB Barcelona) equipped with a quadruple (^1^H, ^13^C, ^15^N, ^31^P) cryogenic resonance probe head and a z-pulse field gradient unit at 298 K using a 1 mM solution of each peptide in the presence or absence of [PdCl_2_(COD)]-1D proton spectra were recorded with a sweep width of 12000 Hz and 32 k data points. A total of 16 scans were accumulated with an acquisition time of 2.05 s. A Watergate w5 composite pulse was used to suppress the water signal. ^1^H 2D-TOCSY and NOESY experiments were acquired in 90% H_2_O/10% D_2_O and used to assign the spin systems corresponding to the peptide resonances.^149−49^ For the 2D-NOESY experiments, mixing times of 300, 150, and 80 ms were acquired to minimize the effect of spin diffusion on the assignments. Spin-locking fields of 8 kHz and 50 ms mixing time were used for the 2D-TOCSY experiments. All 2D spectral widths were 8000 Hz. The data size was 512 points in F1, the indirect dimension, and 2048 points in F2, the direct dimension. For each F1 value, 48 transients were accumulated in the NOESY experiments and 32 in the TOCSY experiments. The data were processed with a combination of exponential and shifted sine-bell window functions for each dimension, followed by automated baseline and phase correction using TopSpin 3.5. The 512 × 2k data matrices were zero-filled to 2k × 2k (NOESY and TOCSY).

### Structure Calculation

To prove that the primary structure (composition and connectivity) corresponds to the theoretical sequence, we have followed the sequence assignment strategy.^49^ We identified the characteristic spin system of every residue in the sequence, using the 2D-TOCSY experiment, and every spin system was connected to the following one via NOEs observed from the side chain of a given residue (*i*) to the amide proton of the following residue (*i* + 1) as well as from the amide proton of (*i*) to the amide of (*i* + 1). The full spin analysis as well as the assignment of the NOEs were carried out manually using CARA software.^50^ Distance restraints derived from the NOESY experiments were used for the NMR-based model building of the peptide in solution using unambiguously assigned peaks exclusively and the program CNS 1.2 (Crystallography and NMR system).^51^ The protocol consisted of an implicit water simulated annealing of 120 structures using 8000 cooling steps followed by an explicit water refinement of the calculated structures using all experimental restraints during 1200 steps. The Pd coordination was not explicitly included in the calculation. To display the metal coordination, we manually optimized the His rotamers to facilitate the coordination and the Pd was added to the final model.

### Computational Studies

*BioMetAll* is a metal-binding prediction tool based on protein and peptide preorganization, considering exclusively the structure and disposition of the backbone.^33^ Several structures were considered to account for variability across WW domain scaffolds. The 12 structures selected were: (1) NMR derived structures with PDB codes 1E0M, 1E0N, 1E0L,^30^ and 1ZR7;^52^ (2) two snapshots of the most representative clusters along a 50 ns cMD for PDB codes 1E0M, 1E0N, and 1ZR7; and (3) two snapshots of the most representative clusters along a 200 ns cMD of the WW0 prototype. BioMetAll was instructed to search for mutations of any of the residues to a His-His motif, avoiding clashes with the backbone and side chains at a distance shorter than 2 Å. Then, results were analyzed accounting for all mutation combinations with several probes that represent more than 40% of the total probes, to ensure that only relevant combinations are considered. The most repeated mutations were in positions 11, 13, 17, 18–20, and 21, suggesting the β1-β2 turn as focal point for the metal binding site.

### Classical Molecular Dynamics

Classical molecular dynamics run for each structure to obtain different snapshots were setup with *xleap*, solvating the peptides with TIP3P water molecules in a box with a distance to the peptide of 10 Å. The system was neutralized with chloride ions. The atoms in the amino acids were represented with the AMBER14SB force field,^41^ while using GAFF force field for the remaining atoms. For the simulations, the OpenMM engine was used, following the OMM Protocol. It starts with an energy minimization of the whole system for 2000 steps, then the water molecules and side chains were heated up from 100 to 300 K. Finally, MD under periodic boundary conditions were run for 50 ns in the case of the crystallographic sequences, and for 200 ns for the WW0 sequence. Convergence of the trajectory was analyzed with *Ptraj* module from AmberTools.

### Pd Coordination Study

The energy calculation of the different coordination modes was carried out at the DFT level of theory using the program Gaussian16 using the hybrid functional B3LYP with the D3 version of Grimme dispersion correction; the basis set used for the C, H, and N atoms was the 6-31+G(d,p), while for the Pd atom, the Stuttgart/Dresden pseudopotential (SDD) was used.^53^ The convergence of forces and step size were considered converged at a tight level (1 × 10^–5^) and the solvent was represented with the solvent-polarizable dielectric continuum model (SMD).^54^ The structures of the models were optimized without any restrictions. The free energy (*G*) values are calculated incorporating the zero-point, thermal and entropy corrections to the potential energy value obtained directly from the SCF calculation and used for comparison between the coordination modes.

### Gaussian Accelerated Molecular Dynamics Study

The metal coordinating parameters were derived from the DFT calculations through the Seminario method through MCPB.py tool,^55^ and the charges were obtained with the restrained electrostatic potential (RESP) model.^56^ The systems with and without Pd were first submitted to a cMD for stabilization and then a total of 26,000,000 steps of different equilibrations. Finally, 400 ns of production of the accelerated molecular dynamics are run, enlarged in the cases where it did not converge up to 500 ns. To assess the conformational properties of the triple-stranded β-sheet, Ramachandran plots were devised for each peptide using the density estimates from the RamachanDraw program, MDtraj,^57^ and NumPy,^58^ to process the data and Matplotlib to design and execute the plots.^59^

### In Vitro Catalytic Studies

The catalytic deprotection of probe **1** to release the uncaged product **2** was performed in a 2 mL HPLC-vial with screw cap. For this purpose, a fresh solution of **1** (10 μL, 20 mM in DMSO, 1.0 equiv) was added to PBS (990 μL), and to the resulting mixture, a solution of the [PdCl_2_(COD)] was added (1 μL, 20 mM in DMSO, 0.1 equiv). The reaction mixture was kept for 24 h at 37 °C under stirring at 1000 rpm. After that time, 50 μL of the reaction was aliquoted, diluted to 100 μL with MeOH, and analyzed by reverse-phase HPLC-MS. The results were treated according to the calibration curve, in which coumarin was used as internal standard. Every value is the average value of two independent measurements.

### Intracellular Reactions

Cells were seeded on glass-bottom plates 48 h before treatment. Culture medium was removed and 300 μL of a 50 μM of probe **1** in FBS-DMEM were added. After 1 h incubation, cells were washed twice with FBS-DMEM, and 300 μL of a 50 μM solution of the palladopeptides in FBS-DMEM were added (the palladopeptides were prepared by mixing the peptides with [PdCl_2_(COD)] (1:1) in water for 1 h before the addition to cells). After a 1 h incubation, cells were washed twice with FBS-DMEM and observed under the microscope with appropriate filters in fresh medium.

## Supporting Information Available

Detailed peptide synthesis, purification, and characterization; spectroscopic studies (CD, NMR); computational studies; and experimental procedures of catalytic and cellular studies (PDF)

## Abbreviations

COD: 1,5-cyclooctadiene
PBS: phosphate-buffered saline
FBS: fetal bovine serum
DMEM: Dulbecco’s modified Eagle medium
TMR: 5-carboxytetramethylrhodamine
