# Correction to “De Novo Engineering of Pd-Metalloproteins and Their Use as Intracellular Catalysts”

**DOI:** 10.1021/jacsau.4c00863

**Published:** 2024-10-03

**Authors:** Soraya Learte-Aymamí, Laura Martínez-Castro, Carmen González-González, Miriam Condeminas, Pau Martin-Malpartida, María Tomás-Gamasa, Sandra Baúlde, José R. Couceiro, Jean-Didier Maréchal, Maria J. Macias, José L. Mascareñas, M. Eugenio Vázquez

In the original Figure 6a, the control image of the peptide **WW13/19** internalization experiment appeared duplicated as the control for peptide **WW19**. This new version of Figure 6 fixes this error and includes the correct microscopy image of the **WW19** internalization control. This correction does not affect in any way the reported results.

**Figure 6:**
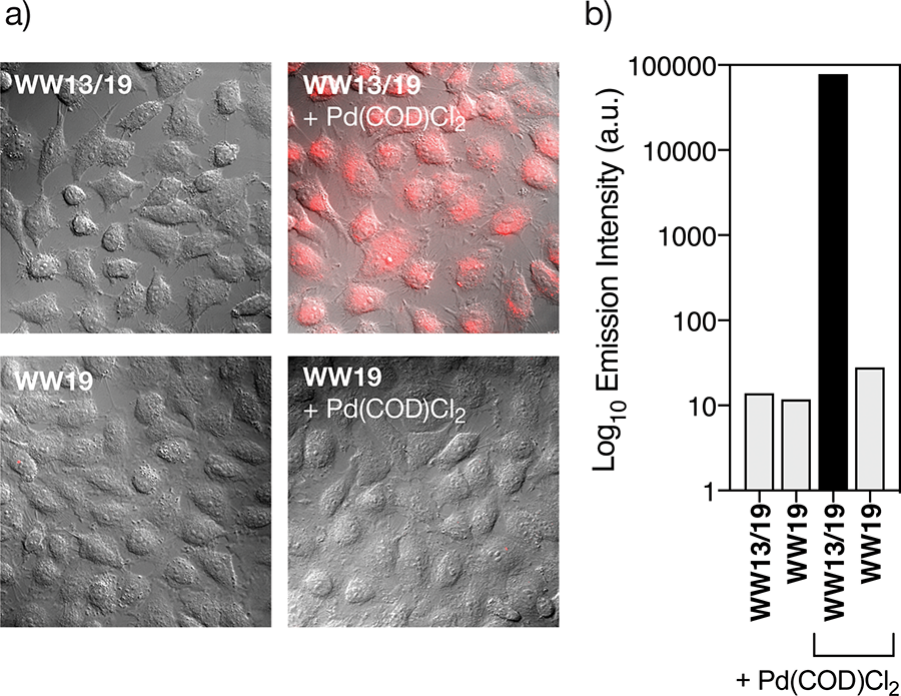
Internalization of TMR-**WW13/19** and its palladoprotein TMR-**WW13/19**[Pd(II)]. (a) Fluorescence microscopy of HeLa cells with 5 μM solutions of the peptides **TMR-WW13/19** and **TMR-WW19** (left) and of their mixtures with [PdCl_2_(COD)] in water (right); (b) semilogarithmic plot of the intracellular TMR emission measured by cell cytometry.

