# Supplementary material for: De Novo Engineering of Pd-Metalloproteins and Their Use as Intracellular Catalysts
Source: JACS Au. 2024 Jun 23;4(7):2630–9. doi: 10.1021/jacsau.4c00379 (PMC11267534; doi:10.1021/jacsau.4c00379)
Supplement: Supplementary file 1 — au4c00379_si_001.pdf [file au4c00379_si_001.pdf]

Supporting Information

De novo Engineering of Pd-metalloproteins and their use as Intracellular Catalysts

Soraya Learte-Aymamí,[a,§] Laura Martínez-Castro,[b,§] Carmen González-González,[a,§] Miriam Condeminas,[c,d] Pau Martin-Malpartida,[c] María Tomás-Gamasa,[a] Sandra Baúlde,[a,†] José R. Couceiro,[a] Jean-Didier Maréchal,[*,b] Maria J. Macias,[*,c,e] José L. Mascareñas,[*,a] M. Eugenio Vázquez[*,a]

[§] These authors contributed equally to this work.

a. Centro Singular de Investigación en Química Bioloxica e Materiais Moleculares (CiQUS), Departamento de Química Orgánica. Universidade de Santiago de Compostela, Santiago de Compostela 15705, Spain.

b. Insilichem, Departament de Química, Universitat Autònoma de Barcelona, Cerdanyola 08193, Spain.

c. Institute for Research in Biomedicine (IRB Barcelona), The Barcelona Institute of Science and Technology (BIST), Baldiri Reixac, 10, Barcelona 08028, Spain.

d. Department of Medicine and Life Sciences, Universitat Pompeu Fabra (MELIS-UPF), Carrer del Doctor Aiguader 88, Barcelona 08003, Spain.

e. Institució Catalana de Recerca i Estudis Avançats (ICREA), Passeig Lluís Companys 23, Barcelona 08010, Spain.

† Current address: CICA – Centro Interdisciplinar de Química e Bioloxía and Departamento de Química. Facultade de Ciencias, Universidade da Coruña. Campus de Elviña, 15071 A Coruña, Spain.

Table 1. Names and sequences of the peptides used in this study. 1E0M corresponds the WW prototype described by Macias *et al*. (*1*). Histidine residues are highlighted in each sequence.

Peptide	Sequence
**1E0M**	SMGLPPGWDE YKTHNGKTYY YNHNTKTSTW TDPRMSS
**WW0**	--GLPPGWTE YKTPNGKTYY YNTNTKTSTW TDPR---
**WW11/13**	--GLPPGWTE **H**K**H**PNGKTYY YNTNTKTSTW TDPR---
**WW11/19**	--GLPPGWTE **H**KTPNGKT**H**Y YNTNTKTSTW TDPR---
**WW13/19**	--GLPPGWTE YK**H**PNGKT**H**Y YNTNTKTSTW TDPR---
**WW9/21**	--GLPPGW**H**E YKTPNGKTYY **H**NTNTKTSTW TDPR---
**WW9/23**	--GLPPGW**H**E YKTPNGKTYY YN**H**NTKTSTW TDPR---
**WW21/23**	--GLPPGWTE YKTPNGKTYY **H**N**H**NTKTSTW TDPR---

General Peptide Synthesis Procedures

All peptide synthesis reagents and amino acid derivatives were purchased from Sigma Aldrich and Iris Biotech; amino acids were purchased as protected Fmoc amino acids with the standard side chain protecting scheme: Fmoc-Ala-OH, Fmoc-Leu-OH, Fmoc-Lys(Boc)-OH, Fmoc-Ser(*t*-Bu)-OH, Fmoc-Glu(O*t*-Bu)-OH, Fmoc-Trp(Boc)-OH, Fmoc-Asn(Trt)-OH, Fmoc-Ile-OH, Fmoc-Thr(*t*-Bu)-OH, Fmoc-Arg(Pbf)-OH, Fmoc-His(Trt)-OH, and Fmoc-Asp(O*t*-Bu)-OH, except for the orthogonally protected Fmoc-Lys(Alloc)-OH, which was purchased from *Bachem*. The resin employed for the SPPS were H-Rink-Amide ChemMatrix (0.57 mmol/g loading) from *Biotage AB.*

Peptides were synthesized following standard Fmoc-peptide synthesis protocols on a 0.1 mmol scale using a 0.5 mmol/g loading *H-Rink* amide *ChemMatrix* resin with a *Liberty Lite* automatic microwave assisted peptide synthesizer from CEM Corporation. The amino acids were coupled with 5-fold excess DIC as activator, Oxime as base and DMF as solvent. Couplings were conducted for 4 min at 90 °C. Deprotection of the temporal Fmoc protecting group was performed by treating the resin with 20% piperidine in DMF for 1 min at 75 °C.

Synthesis of labeled TMR-**WW13/19** and TMR-**WW19.** Once the peptides were fully assembled and still attached to the solid support, we coupled Fmoc-6-aminohexanoic acid (Fmoc-Ahx-OH) as spacer using standard solid-phase methods (activation with 1.0 equiv. of HATU in 2 mL DMF with 3 mL 0.195 M DIEA/DMF). The terminal Fmoc protecting group was removed (20% piperidine/DMF) and a mixture of 5-carboxytetramethylrhodamine (TMR-OH, 3.0 equiv., 0.15 mmol, 64.5 mg), 3 equiv. of HATU and 5 equiv. of DIEA 0.195 M in DMF was added onto the resin and shaken for 60 min.

Cleavage/deprotection. The resin-bound peptide was treated for 2h with the following cleavage cocktail: 900 µL TFA, 50 µL CH_2Cl_2, 25 µL H_2O and 25 µL TIS (1 mL of cocktail / 40 mg resin). The resin was filtered, and the cocktail was added onto ice-cold diethyl ether. After 10 - 30 min, the precipitate was centrifuged and washed again with of ice-cold ether. The solid residue was dried under argon and re-dissolved in water.

The compounds were analyzed by analytical **UHPLC-MS** with an *Agilent 1200* series LC/MS using a *SB C_{18}* (1.8 µm, 2.1 × 50 mm) analytical column from *Phenomenex*. Standard conditions for analytical UHPLC consisted on a linear gradient from 5 to 95% of solvent B for 20 min at a flow rate of 0.35 mL/min (A: water with 0.1% TFA, B: acetonitrile with 0.1% TFA). Compounds were detected by UV absorption at 222, 270, and 330 nm. Electrospray Ionization Mass Spectrometry (ESI/MS) was performed with an *Agilent 6120 Quadrupole* LC/MS model in positive scan mode using direct injection of the purified peptide solution into the MS detector.

Purification of the peptides was performed on a semipreparative RP-HPLC with an *Agilent* 1100 series LC equipped with a UV-visible detector using a *Phenomenex Luna–C_{18}* (250 × 10 mm) reverse-phase column. Standard conditions for purification by RP–HPLC consisted on a linear gradient 5 to 75% B over 40 min at a flow rate of 4 mL/min. (A: H_2O 0.1% TFA, B: CH_3CN 0.1% TFA). Collected fractions with pure products were lyophilized with a *ThermoSavant Modulyo D* freeze drier equipped with an *Edwars RV* high vacuum pump.

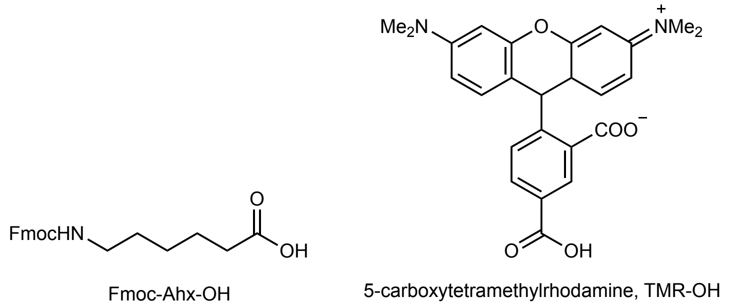

Figure S1. Structures of Fmoc-Ahx-OH, and 5-carboxytetramethylrhodamine (TMR-OH).

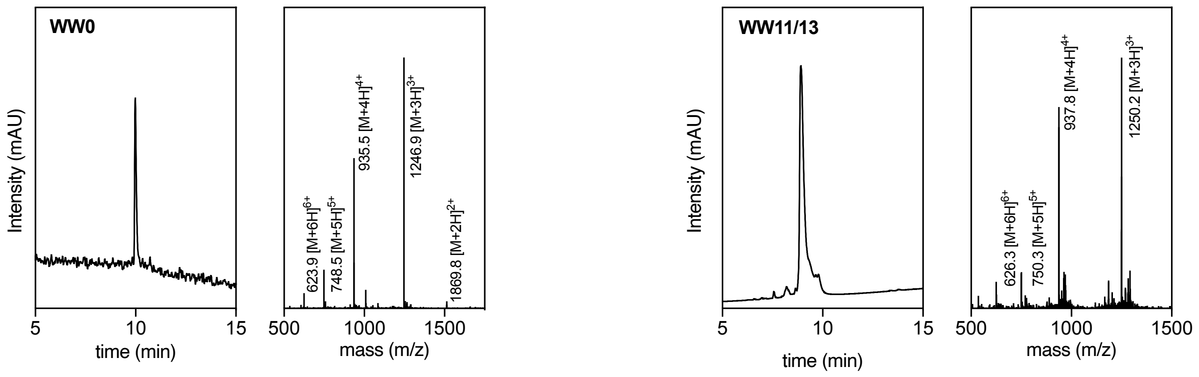

Figure S2. HPLC traces of the purified peptides used in this study and their corresponding ESI-MS spectra showing in each case peaks matching the expected masses.

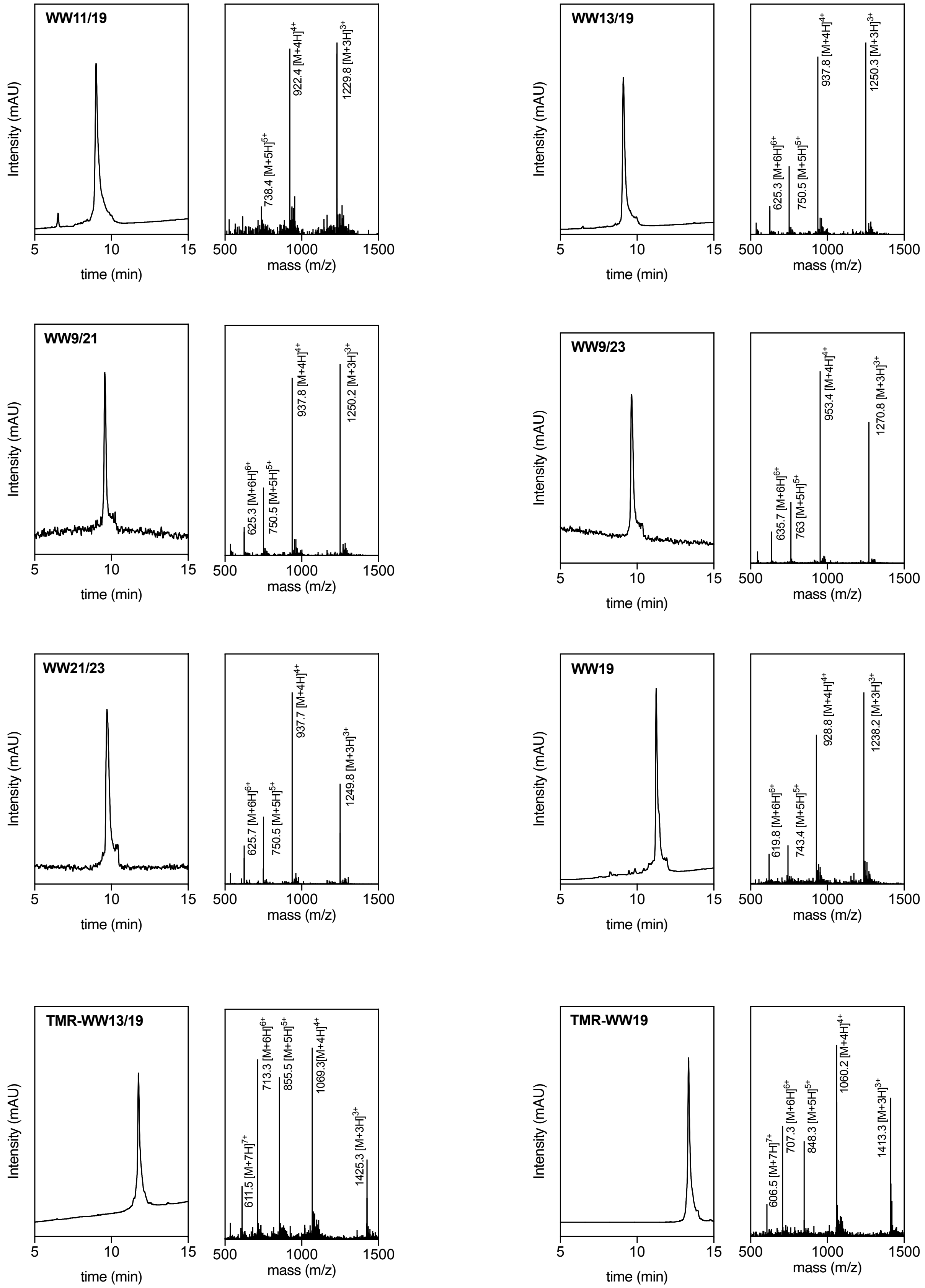

Figure S2 (cont.). HPLC traces of the purified peptides used in this study and their corresponding ESI-MS spectra showing in each case peaks matching the expected masses.

Coordination to palladium. Peptides (1 mM) were mixed with equimolar amounts of [PdCl$_2$(COD)] in H_2O and the resulting mixtures were analyzed after 15 min. by Electrospray Ionization Mass Spectrometry (ESI/MS) with an *Agilent 6120 Quadrupole* LC/MS model in positive scan mode using direct injection of the purified peptide solution into the MS detector. The peaks corresponding to the apo-peptides are shown in black, and those corresponding to the complexes, that show the mass of the peptide and Pd(II), are in blue.

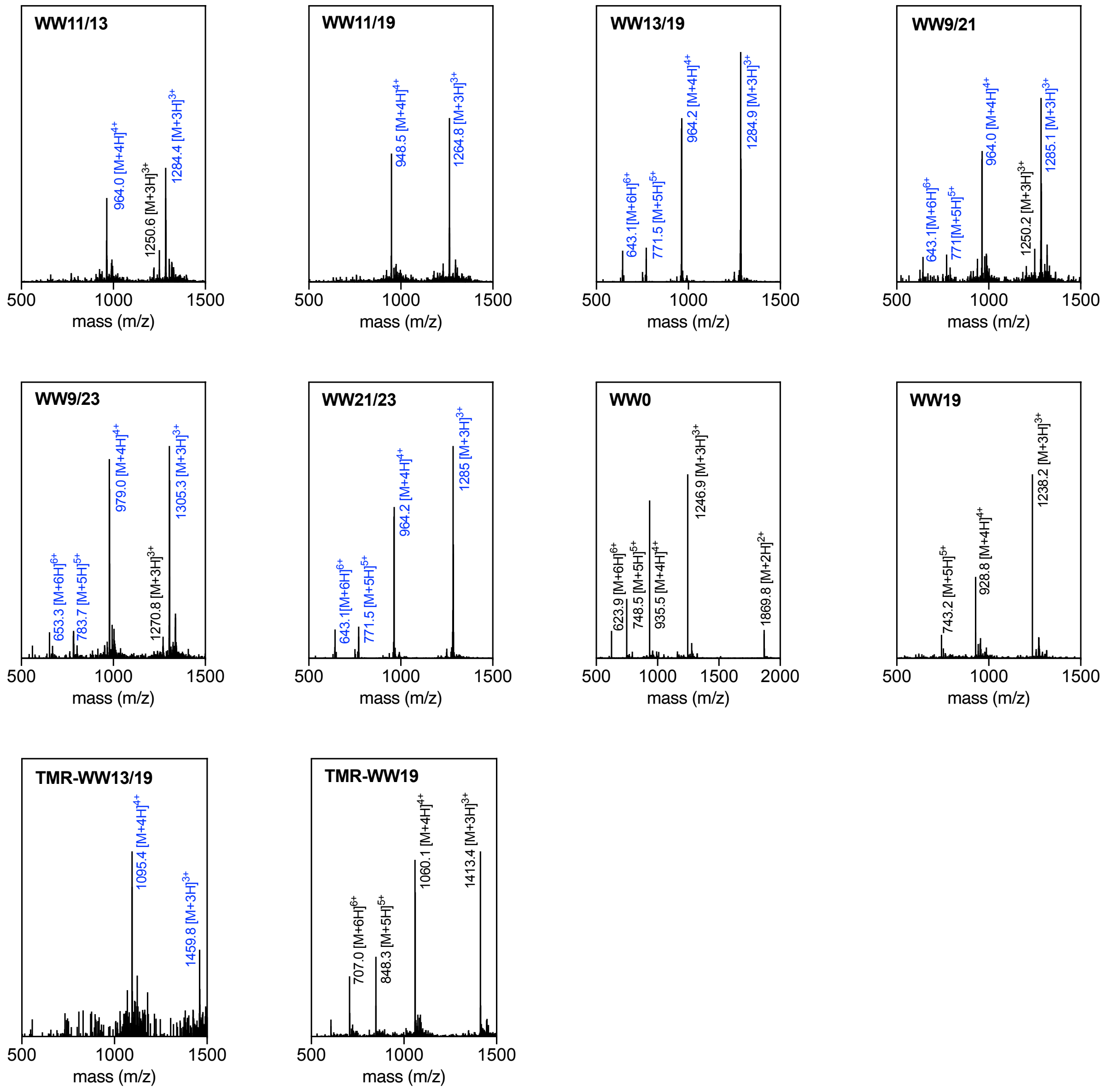

Figure S3. ESI-MS spectra of the mixture of each peptide (1 mM) with 1.0 equiv. of [PdCl$_2$(COD)] in water after mixing for 15 min. The peaks corresponding to the apo-peptides are labeled in black, those of the corresponding palladopeptides, are in blue. Only the peptides featuring pairs of His show effective coordination, so that control peptides **WW0**, **WW19** and **TMR-WW19**, that feature only one histidine, do not show peaks corresponding to their coordination products.

Synthesis and characterization of probes 1 and 3

The synthesis was made according to a previously published procedure (*2*).

General protocols: Chemical were purchased from *Sigma Aldrich*, *Alfa Aesar*, and *Iris Biotech* and used without further purification. Reaction mixtures were stirred using Teflon-coated magnetic stir bars. The abbreviation "rt" refers to reactions carried out at room temperature (approximately at 23 °C). The temperature was maintained using Thermowatch-controlled heating blocks. Thin-layer chromatography (TLC) was performed on silica gel plates (*Merck 60 silica gel F254*) and the reaction components were visualized by observation under UV light and/or by treating the plates with $KMnO_4$ or ninhydrine followed by heating. Flash chromatography was carried out on silica gel (*Merck Geduran Su 60*, 40 - 63 µM silica gel, normal phase). Concentration refers to the removal of volatile solvents via distillation using a rotary evaporator *Büchi R-210* equipped with a *thermostated bath B-491*, a vacuum regulator *V-850,* followed by residual solvent removal under high vacuum.

Measurements of **fluorescence** were performed using a Varian Cary Eclipse fluorimeter thermostated cell compartment at 20 ± 0.5 °C using 1 cm quartz cells. The measurements were made with the following settings: increment 1.0 nm, averaging time 0.1 s, excitation slit width 5.0 nm, emission slit width 10.0 nm, PMT voltage 700 V.

Measurements of **UV/Vis** were made in a Jasco V-630 spectrophotometer coupled to a Jasco ETC-717 temperature controller, using a standard Hellma semi-micro cuvette (108.002-QS) with a light path of 10 mm. Measurements were made at 20 °C. Acquisition parameters were: 220-700 nm range, scan speed of 200 nm/min, resolution of 0.2 nm.

Abbreviations: DIC: N,N′-Diisopropylcarbodiimide; DMF: *N,N*-dimethylformamide; DIEA: *N,N*-diisopropyl-ethylamine; HATU: 2-(1*H*-7-aza- benzotriazol-1-*yl*)-1,1,3,3-tetramethyluronium hexafluoro-phosphate; TFA: trifluoroacetic acid; TIS: triisopropyl silane; TMR: tetramethylrhodamine dye

^{1}H- and ^{13}C NMR experiments were carried out using a *Varian Mercury* 300 MHz NMR spectrometer. Chemical shift values are reported in ppm with the solvent resonance as the internal standard ($CDCl_3$: δ 7.26 for ^{1}H, δ 77.2 for ^{13}C; DMSO: δ 2.50 for ^{1}H, δ 39.5 for ^{13}C). Coupling constants *J* are given in Hertz (Hz). Multiplicities are reported as follows: s = singlet, d = doublet, t = triplet, q = quartet, m = multiplet or as a combination of them. Coupling constants in Hertz (Hz). The chemical shifts for protons (δ) are reported in parts per million downfield from tetramethyl silane and are referenced to residual protium in the NMR solvent ($CHCl_3$ δ = 7.26). Chemical shifts for carbon are reported in parts per million downfield from tetramethyl silane and are referenced to the carbon resonances of the solvent ($CDCl_3$ δ = 77.0). NMR spectra were analyzed using *MestreNova* NMR data processing software (*www.mestrelab.com*).

Synthesis of probe 1

The synthesis was made according to a previously published procedure (*2*).

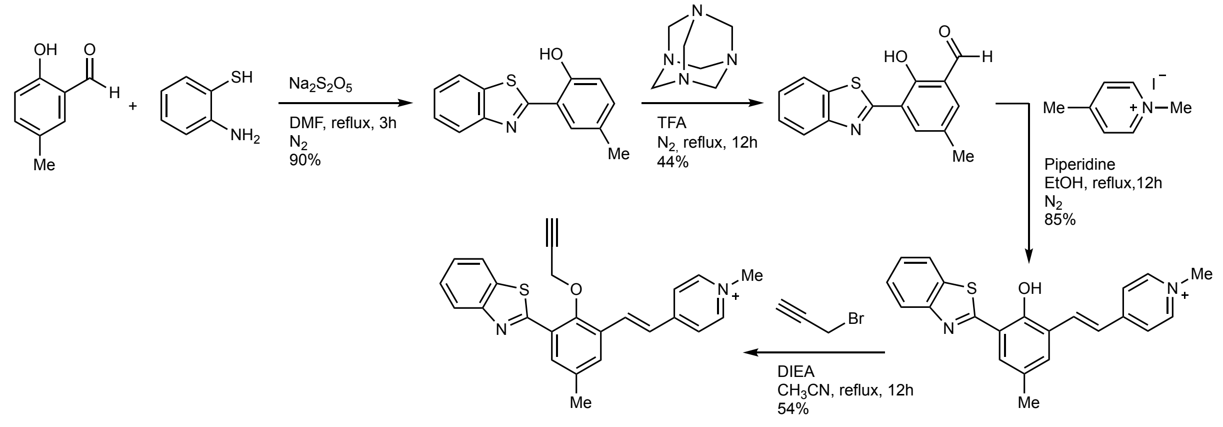

Scheme S1. Synthetic route to obtain (E)-4-(3-(benzo[d]thiazol-2-yl)-5-methyl-2-(prop-2-yn-1-yloxy)styryl)-1-methylpyridin-1-ium (**1**).

2-(benzo[d]thiazol-2-*yl*)-4-methylphenol. 2-hydroxy-5-methylbenzaldehyde (1.50 g, 11.0 mmol, 1.1 equiv.) and $Na_2S_2O_5$ (1.80 g, 9.5 mmol, 1.0 equiv.) were dissolved in 15 mL anhydrous DMF in a Schlenk flask under an inert atmosphere. 2-aminobenzenethiol (1.20 ml, 11.0 mmol, 1.1 equiv.) was added, and the reaction was then stirred at 110 °C for 3 h. 10 mL mili-Q water were added to the reaction mixture leading to the formation of a white precipitate. The precipitate was collected by filtration to afford the desired compound as a white powder (2.57 g, 90%). **^{1}H NMR** (300 MHz, $CDCl_3$) δ 12.28 (s, 1H), 7.96 (d, *J* = 8.1 Hz, 1H), 7.87 (d, *J* = 7.9 Hz, 1H), 7.54 – 7.32 (m, 3H), 7.18 (d, *J* = 8.6 Hz, 1H), 7.01 (d, *J* = 8.5 Hz, 1H), 2.35 (s, 4H). **^{13}C NMR** (75 MHz, $CDCl_3$) δ 169.5, 155.9, 152.0, 133.8, 132.7, 128.7, 128.4, 126.7, 125.5, 122.2, 121.5, 117.7, 116.4, 20.5. **HLPC-MS (ESI):** calcd.: $C_{14}H_{11}NOS$ $[M]^+$: 241.1, found: 242.15.

3-(benzo[d]thiazol-2-*yl*)-2-hydroxy-5-methylbenzaldehyde. 2-(benzo[d]thiazol-2-*yl*)-4-methylphenol (2.57 g, 10.7 mmol, 1 equiv.) and hexamethylenetetramine (4.51 g, 32.0 mmol, 3.0 equiv.) were dissolved in TFA (18 mL) under inert atmosphere, the reaction was heated at reflux overnight. 4 M HCl (180 mL) was added to the reaction mixture and the resulting mixture was extracted with Et_2O (2 × 150 mL). The organic layer was dried wit $MgSO_4$, filtered, and the solvent evaporated. The crude was redissolved in cold Et_2O and to afford the desired compound as a white solid (1.28 g, 44%). **^{1}H NMR** (300 MHz, $CDCl_3$) δ 10.47 (s, 1H), 8.03 (d, *J* = 8.1 Hz, 1H), 7.93 (m, 2H), 7.70 (s, 1H), 7.54 (t, *J* = 7.2 Hz, 1H), 7.44 (t, *J* = 7.6 Hz, 1H), 2.40 (s, 3H).**^{13}C NMR** (75 MHz, $CDCl_3$) δ 191.2, 167.0, 158.7, 151.5, 135.4, 133.3, 132.9, 129.1, 127.0, 126.0, 123.8, 122.5, 121.7, 118.85, 20.4. **HLPC-MS (ESI):** calcd.: $C_{15}H_{11}NO_2S$ $[M]^+$: 269.1, found: 270.1.

***(E)*-4-(3-(benzo[d]thiazol-2-*yl*)-2-hydroxy-5-methylstyryl)-1-methylpyridin-1-ium (2).** 3-(benzo[d]thiazol-2-*yl*)-2-hydroxy-5-methylbenzaldehyde (1.00 g, 3.70 mmol, 1.0 equiv.), 1,4-dimethylpyridinium and piperidine (385.0 µL, 3.70 mmol, 1.0 equiv.) were dissolved in EtOH (75 mL) and refluxed overnight under N_2 atmosphere. The solvents were removed under vacuum and the resulting solid is redissolved in the minimal amount of MeOH and precipitated with cold Et_2O to afford the compound (**2**) after filtration as an orange solid (1.52 g, 85%). **^{1}H NMR** (300 MHz, DMSO) δ 13.05 (s, 1H), 8.84 (d, *J* = 6.9 Hz, 2H), 8.21 (d, *J* = 6.9 Hz, 3H), 8.16 – 8.04 (m, 2H), 7.76 (d, *J* = 9.6 Hz, 2H), 7.69 – 7.46 (m, 3H), 4.27 (s, 3H), 2.38 (s, 3H). **^{13}C NMR** (75 MHz, DMSO) δ 168.2, 154.0, 152.6, 150.7, 145.0, 134.7, 132.4, 130.6, 128.9, 127.1, 126.0, 124.3, 123.5, 123.4, 122.3, 121.9, 117.0, 46.9, 19.9. **HLPC-MS (ESI):** calcd.: $C_{22}H_{19}NOS^+$ $[M]^+$: 359.1, found: 359.2.

(*E*)-4-(3-(benzo[d]thiazol-2-yl)-5-methyl-2-(prop-2-yn-1-yloxy)styryl)-1-methylpyridin-1-ium (1). To a solution of compound **2** (972.0 mg, 2.0 mmol, 1.0 equiv.) in CH_3CN (7 mL) propargyl bromide (350. 0 µL, 4.0 mmol, 2.0 equiv.) and DIEA (700.0 µL, 4.0 mmol, 2.0 equiv.) were added, and the reaction mixture was refluxed overnight. Cold MeOH was added, and the precipitate was collected by filtration. Compound (**1**) was obtained as bright yellow solid after washing with MeOH (583.0 mg, 61%) (*3*). **^{1}H NMR** (300 MHz, DMSO) δ 8.70 (d, *J* = 6.7 Hz, 2H), 8.06 (d, *J* = 7.1 Hz, 2H), 8.01 (s, 1H), 7.95 (d, *J* = 7.9 Hz, 1H), 7.91 – 7.80 (m, 2H), 7.68 (s, 1H), 7.46 – 7.31 (m, 2H), 7.26 (t, *J* = 7.7 Hz, 1H), 4.59 (s, 2H), 4.08 (s, 3H), 3.08 (s, 1H), 2.24 (s, 3H). **^{13}C NMR** (75 MHz, DMSO) δ 161.9, 152.2, 152.0, 151.7, 145.2, 135.5, 135.0, 133.9, 131.0, 130.6, 129.8, 127.4, 126.4, 125.5, 125.4, 123.9, 122.7, 122.0, 80.2, 78.1, 62.8, 47.0, 20.4. **HLPC-MS (ESI):** calcd.: $C_{25}H_{21}N_2OS^+$ $[M]^+$: 397.2 $[M+2H]^{2+}$: 199.2, found: 397.2, 199.2.

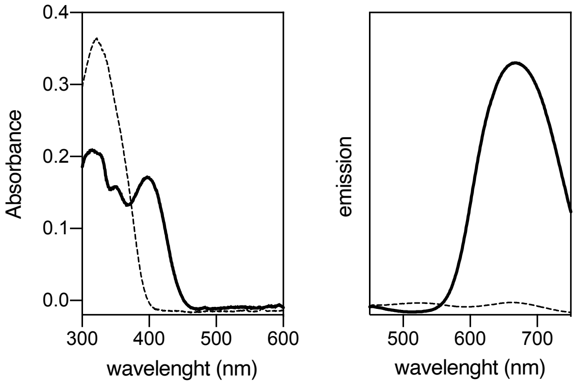

Figure S4. Left: Absorbance spectra of the propargylated fluorogenic probe **1** (dashed line) and the deprotected product **2** (thick solid line). Right: fluorescence emission spectra of **1** (dashed line) and **2** (thick solid line). Measurements taken at 20 µM, 7:3 v/v DMSO/H_2O, λ_{exc} = 400 nm.

Synthesis of probe 3

The synthesis was made according to a previously published procedure (*2*, *4*).

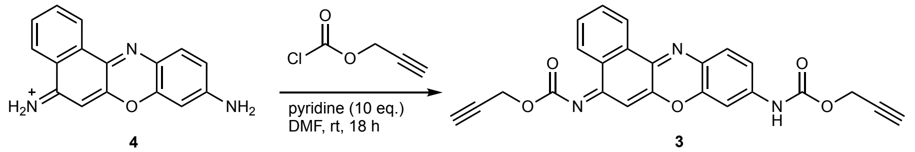

Scheme S2. Synthesis of the propargylated cresyl violet probe **3**.

Prop-2-yn-1-*yl* (*E*)-(5-(((prop-2-yn-1-yloxy)carbonyl)imino)-5H-benzo[a]phenoxazin-9-yl)carbamate (3). Cresyl violet perchlorate (100 mg, 0.27 mmol) was dissolved in anhydrous DMF (1.0 mL) and cooled to 0 °C. Separately, pyridine (140 µL, 2.8 mmol, 10.0 equiv.) and propargyl chloroformate (116 µL, 1.4 mmol, 5 equiv.) were each dissolved in anhydrous DMF (0.5 mL) and the solutions added dropwise to the cresyl violet. The reaction mixture was stirred at room temperature for 18 h, after which it was diluted with iPrOH/$CHCl_3$ (25:75, 10.0 mL), washed with 5% HCl (2 × 10.0 mL) and $NaHCO_3$ (2 × 5.0 mL) and dried over $MgSO_4$. The solvent was removed in vacuo and the resulting crude purified by silica gel column chromatography using a hexane/ethyl acetate (2:1) mixture. The compound **4** was obtained as an orange solid (21%). **^{1}H NMR** (300 MHz, DMSO-d^6) δ 10.46 (s, 1H), 8.58 (d, *J* = 9.3 Hz, 1H), 8.30 (d, *J* = 9.4 Hz, 1H), 7.93 – 7.67 (m, 3H), 7.58 (s, 1H), 7.42 (d, *J* = 8.7 Hz, 1H), 4.87 (dd, *J* = 26.4, 2.4 Hz, 4H), 3.63 (dt, *J* = 15.6, 2.4 Hz, 2H). **^{13}C NMR** (75 MHz, DMSO-d^6) δ 162.1, 159.3, 152.3, 148.6, 144.3, 142.2, 131.7, 131.3, 130.9, 129.9, 128.4, 124.8, 124.1, 115.3, 103.8, 100.1, 78.6, 78.5, 77.9, 53.6, 52.5.

2-(benzo[d]thiazol-2-yl)-4-methylphenol NMR characterization:

^{1}H NMR (300 MHz, $CDCl_3$)

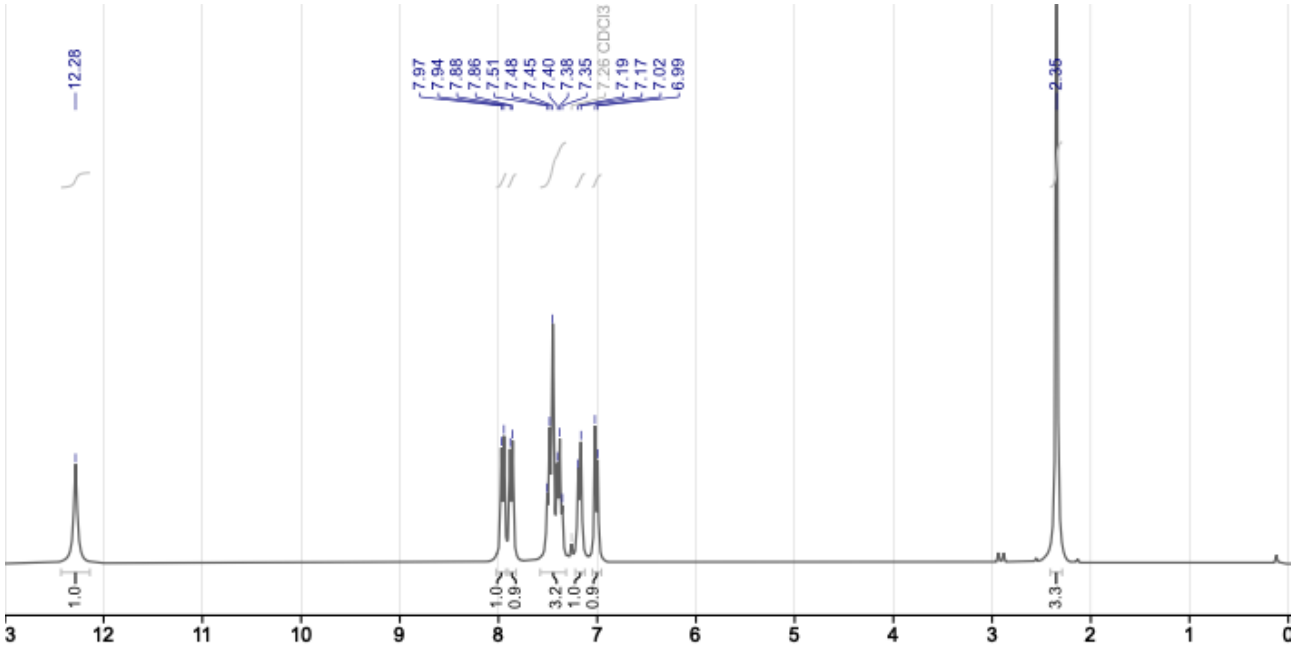

^{13}C NMR (75 MHz, $CDCl_3$)

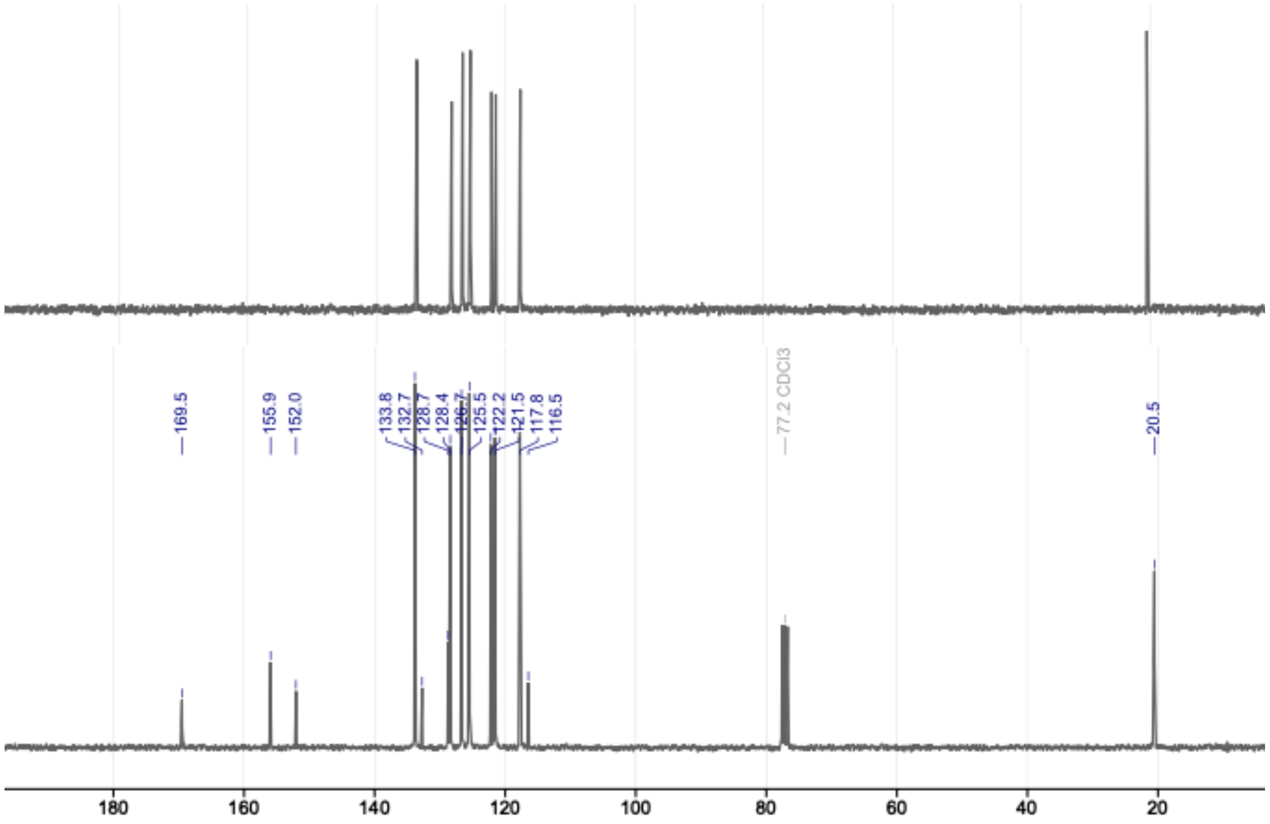

^{1}H NMR (300 MHz, $CDCl_3$) δ 12.28 (s, 1H), 7.96 (d, *J* = 8.1 Hz, 1H), 7.87 (d, *J* = 7.9 Hz, 1H), 7.54 – 7.32 (m, 3H), 7.18 (d, *J* = 8.6 Hz, 1H), 7.01 (d, *J* = 8.5 Hz, 1H), 2.35 (s, 4H). **^{13}C NMR** (75 MHz, $CDCl_3$) δ 169.5, 155.9, 152.0, 133.8, 132.7, 128.7, 128.4, 126.7, 125.5, 122.2, 121.5, 117.7, 116.4, 20.5.

Figure S5. ^{1}H and ^{13}C NMR characterization of 2-(benzo[d]thiazol-2-yl)-4-methylphenol in $CDCl_3$.

3-(benzo[d]thiazol-2-yl)-2-hydroxy-5-methylbenzaldehyde NMR characterization:

^{1}H NMR (300 MHz, $CDCl_3$)

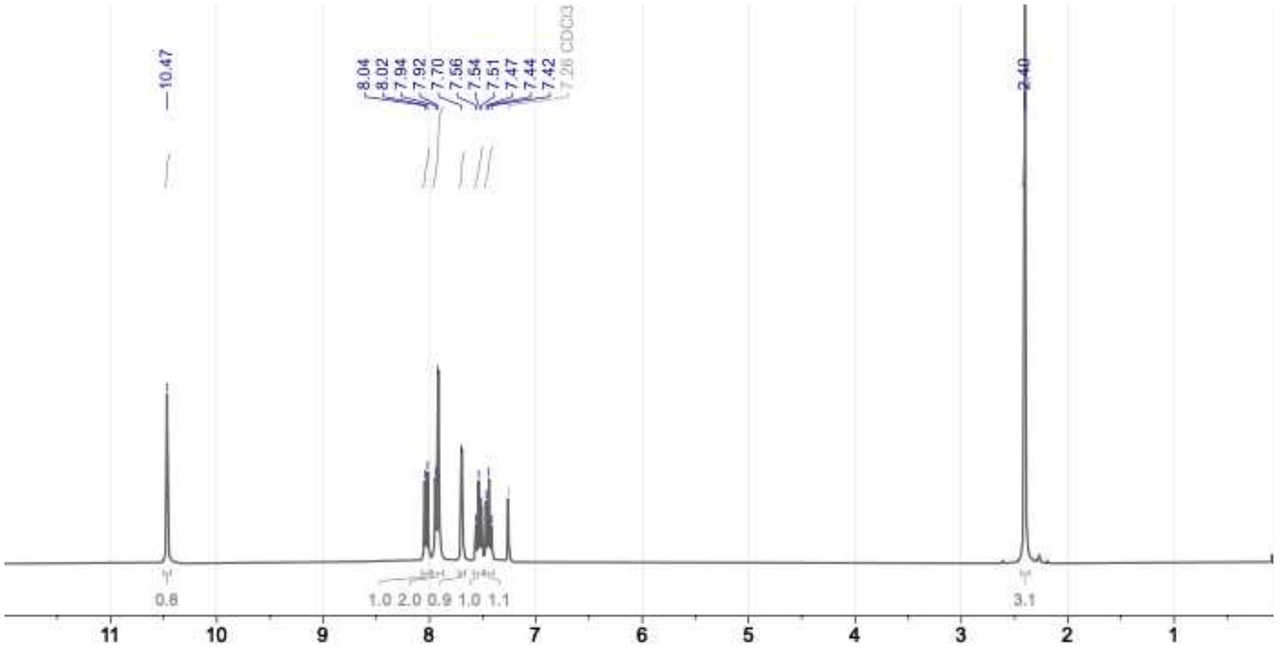

^{13}C NMR (75 MHz, $CDCl_3$)

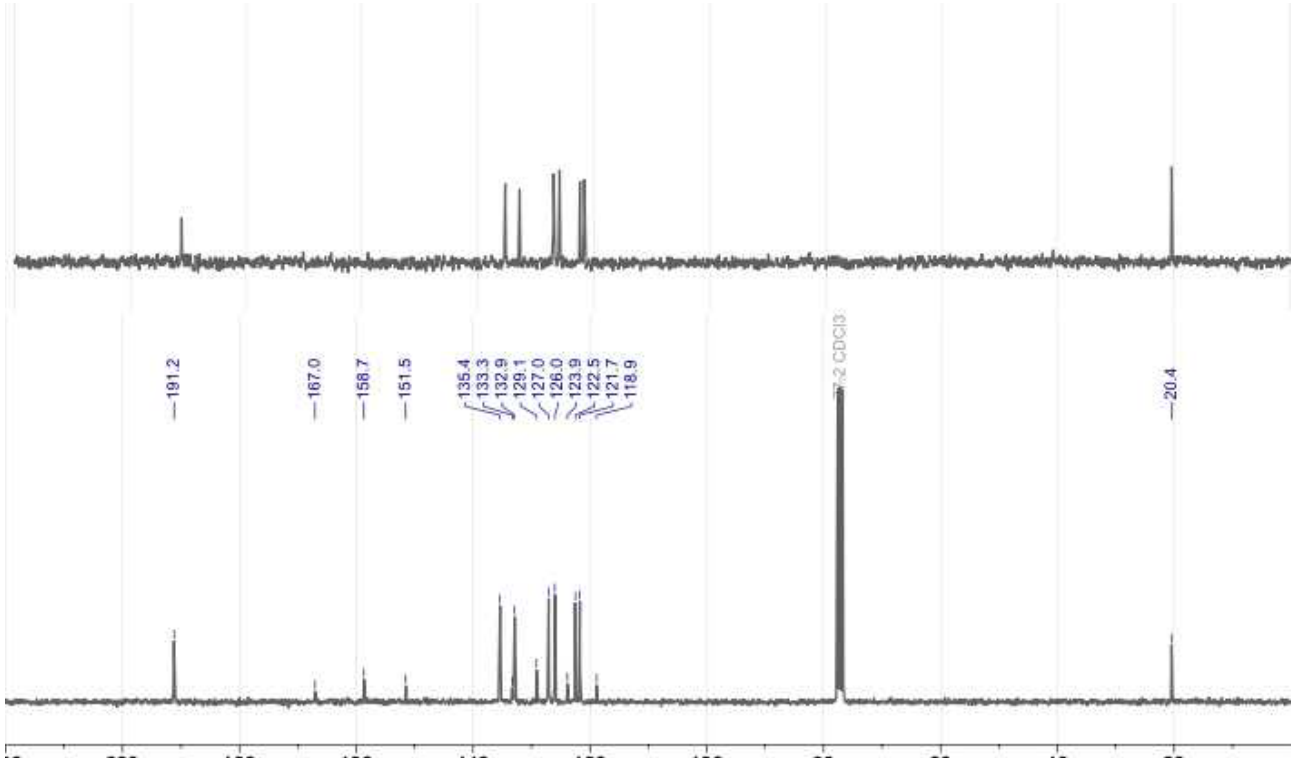

^{1}H NMR (300 MHz, $CDCl_3$) δ 10.47 (s, 1H), 8.03 (d, *J* = 8.1 Hz, 1H), 7.93 (m, 2H), 7.70 (s, 1H), 7.54 (t, *J* = 7.2 Hz, 1H), 7.44 (t, *J* = 7.6 Hz, 1H), 2.40 (s, 3H).**^{13}C NMR** (75 MHz, $CDCl_3$) δ 191.2, 167.0, 158.7, 151.5, 135.4, 133.3, 132.9, 129.1, 127.0, 126.0, 123.8, 122.5, 121.7, 118.85, 20.4.

Figure S6. ^{1}H and ^{13}C NMR characterization of 3-(benzo[d]thiazol-2-yl)-2-hydroxy-5-methylbenzaldehyde in $CDCl_3$.

Probe 2 NMR characterization:

^{1}H NMR (300 MHz, DMSO)

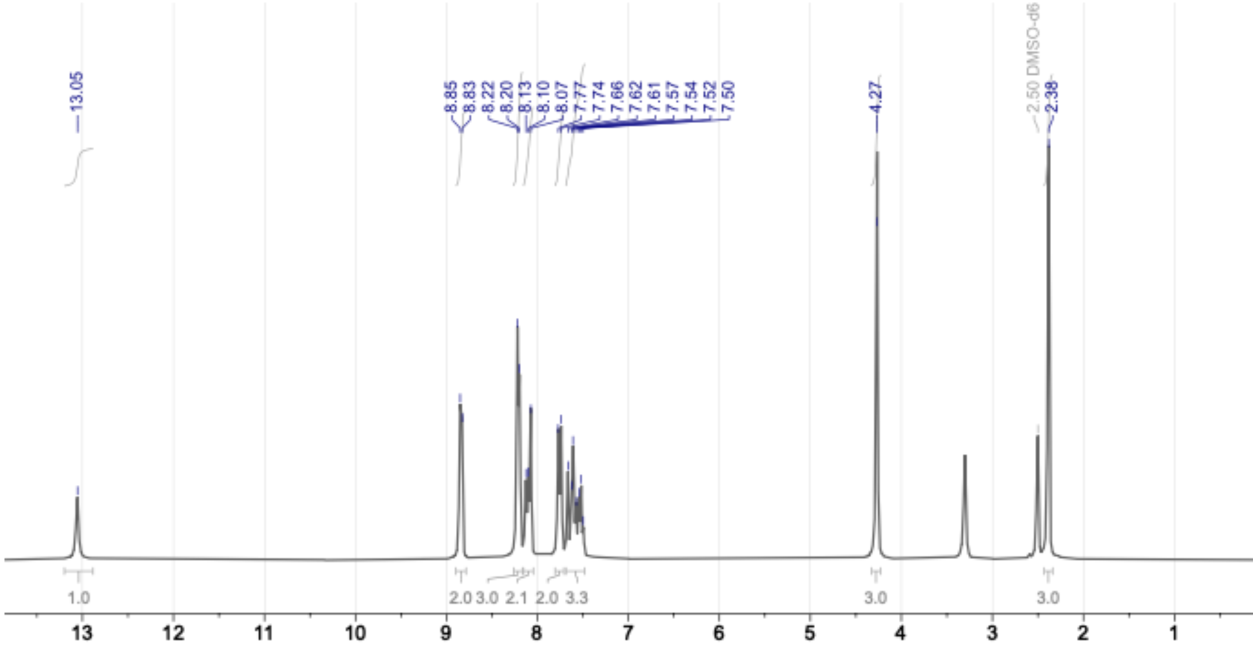

^{13}C NMR (75 MHz, DMSO)

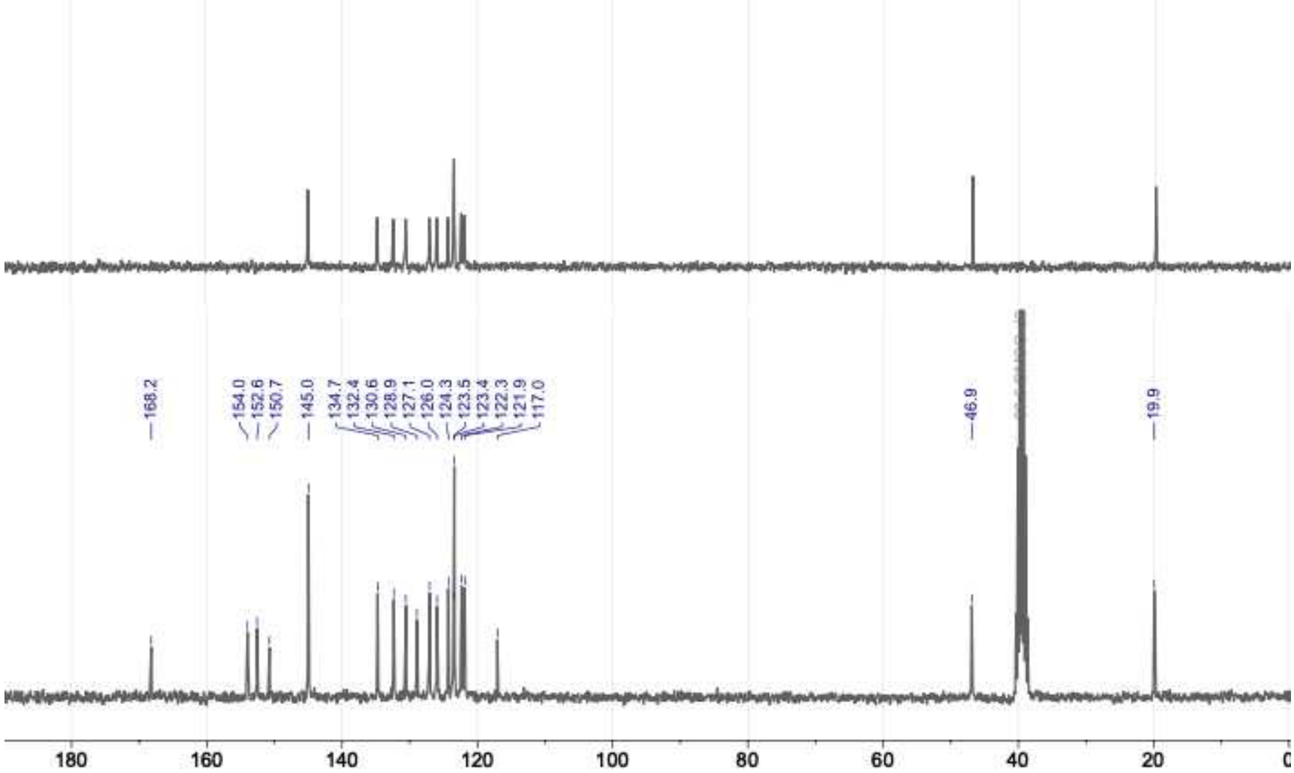

^{1}H NMR (300 MHz, DMSO) δ 13.05 (s, 1H), 8.84 (d, J = 6.9 Hz, 2H), 8.21 (d, J = 6.9 Hz, 3H), 8.16 – 8.04 (m, 2H), 7.76 (d, J = 9.6 Hz, 2H), 7.69 – 7.46 (m, 3H), 4.27 (s, 3H), 2.38 (s, 3H). **^{13}C NMR** (75 MHz, DMSO) δ 168.2, 154.0, 152.6, 150.7, 145.0, 134.7, 132.4, 130.6, 128.9, 127.1, 126.0, 124.3, 123.5, 123.4, 122.3, 121.9, 117.0, 46.9, 19.9.

Figure S7. ^{1}H and ^{13}C NMR characterization of (E)-4-(3-(benzo[d]thiazol-2-yl)-2-hydroxy-5-methylstyryl)-1-methylpyridin-1-ium, HBTP’ (**2**) in DMSO.

Probe 1 NMR characterization:

^{1}H NMR (300 MHz, DMSO)

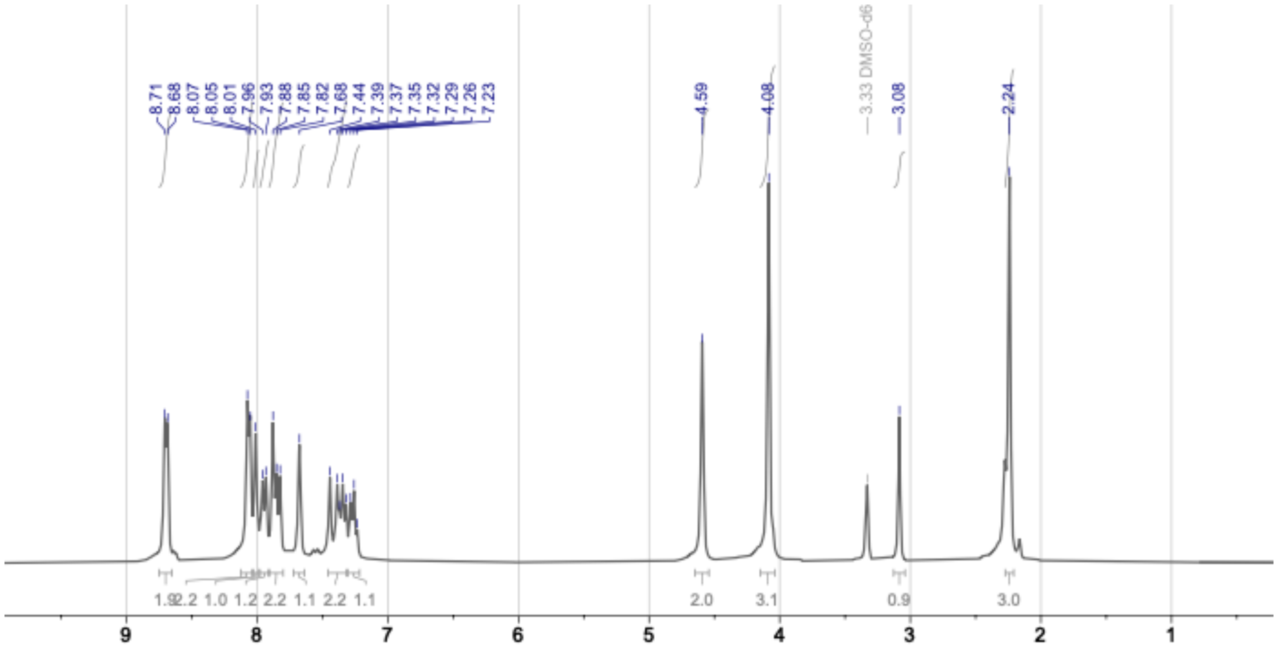

^{13}C NMR (75 MHz, DMSO)

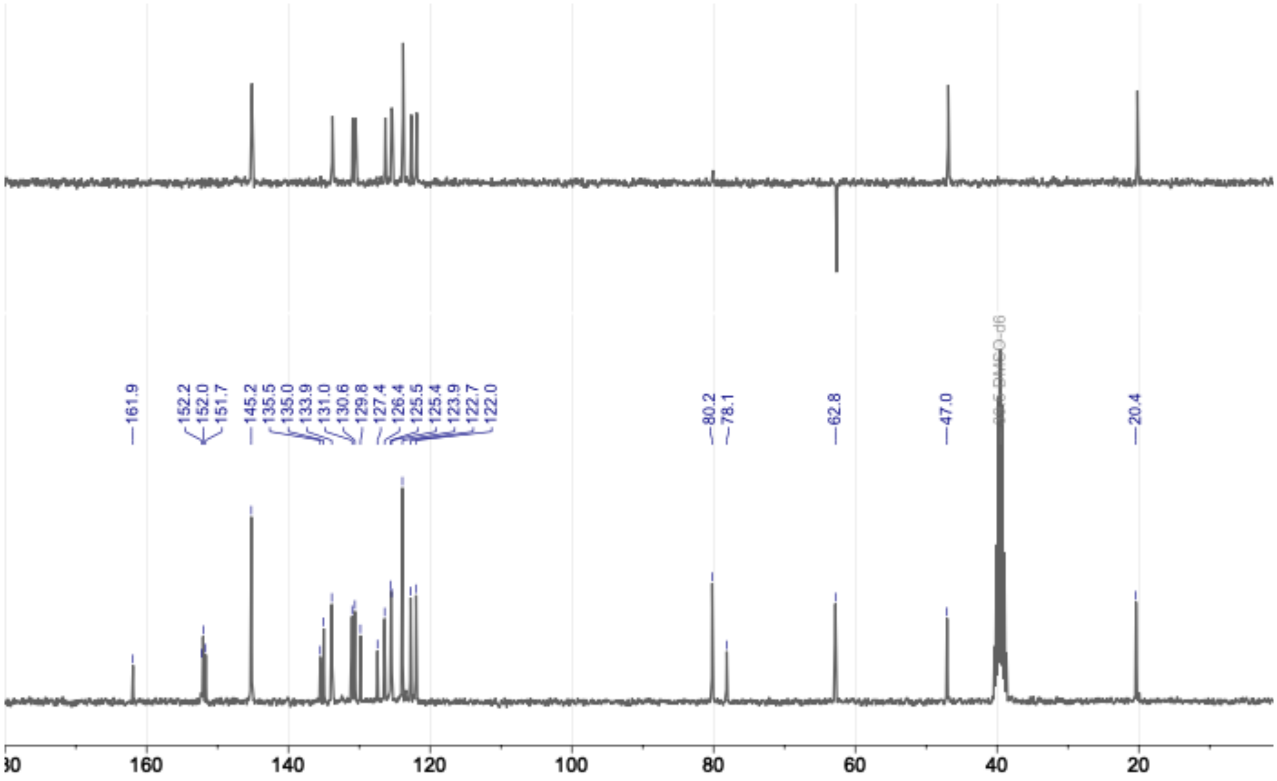

^{1}H NMR (300 MHz, DMSO) δ 8.70 (d, *J* = 6.7 Hz, 2H), 8.06 (d, *J* = 7.1 Hz, 2H), 8.01 (s, 1H), 7.95 (d, *J* = 7.9 Hz, 1H), 7.91 – 7.80 (m, 2H), 7.68 (s, 1H), 7.46 – 7.31 (m, 2H), 7.26 (t, *J* = 7.7 Hz, 1H), 4.59 (s, 2H), 4.08 (s, 3H), 3.08 (s, 1H), 2.24 (s, 3H). **^{13}C NMR** (75 MHz, DMSO) δ 161.9, 152.2, 152.0, 151.7, 145.2, 135.5, 135.0, 133.9, 131.0, 130.6, 129.8, 127.4, 126.4, 125.5, 125.4, 123.9, 122.7, 122.0, 80.2, 78.1, 62.8, 47.0, 20.4.

Figure S8. ^{1}H and ^{13}C NMR characterization of (E)-4-(3-(benzo[d]thiazol-2-yl)-5-methyl-2-(prop-2-yn-1-yloxy)styryl)-1-methylpyridin-1-ium (**1**) in DMSO.

Probe 3 NMR characterization:

^{1}H NMR (300 MHz, DMSO)

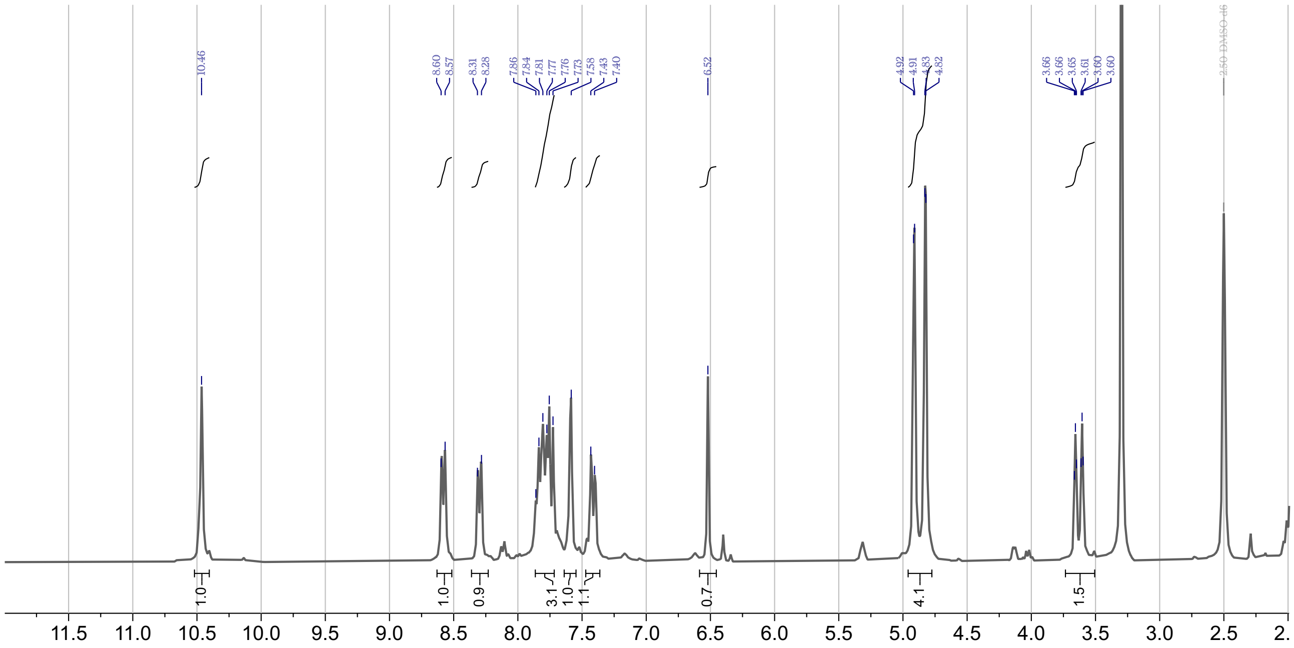

^{13}C NMR (75 MHz, DMSO)

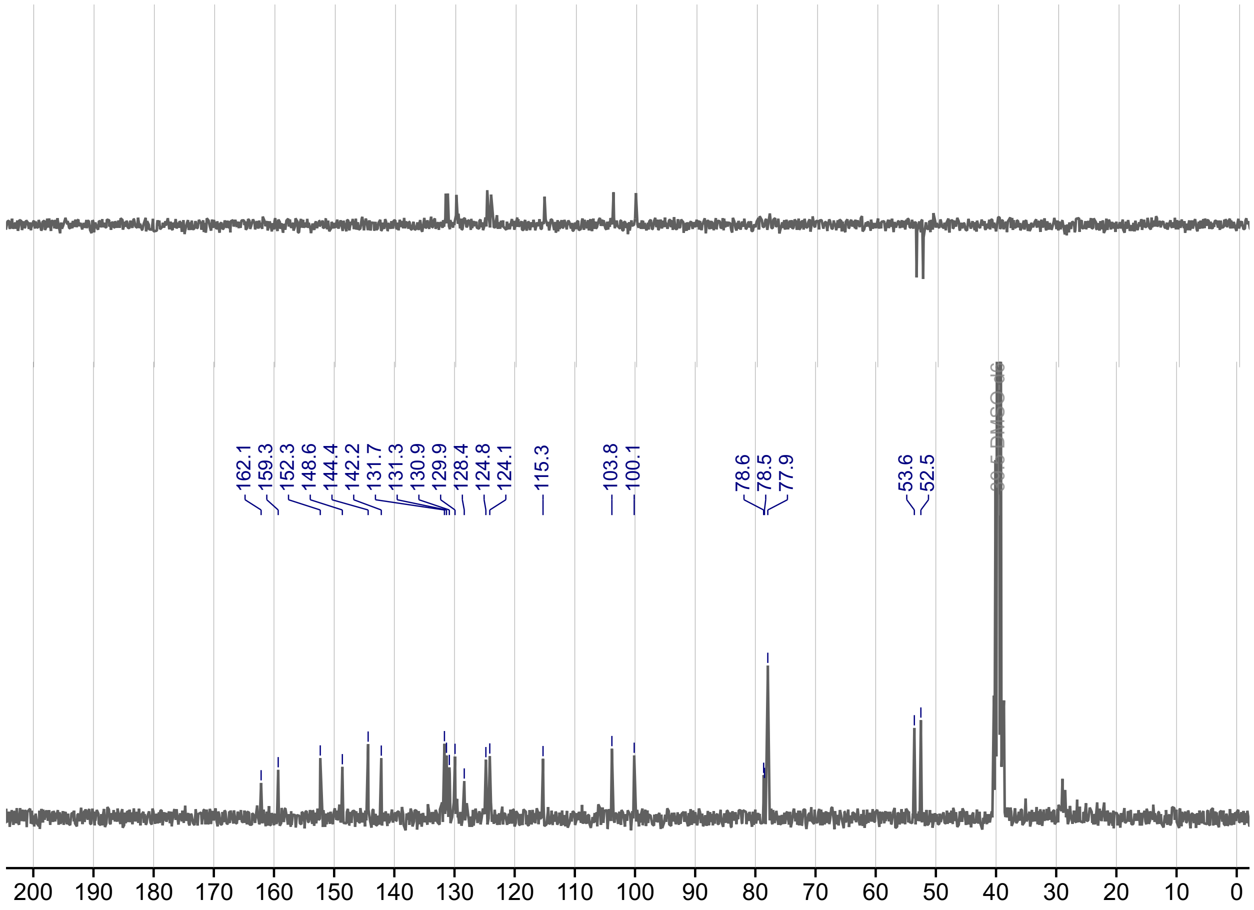

^{1}H NMR (300 MHz, DMSO-d^6) δ 10.46 (s, 1H), 8.58 (d, *J* = 9.3 Hz, 1H), 8.30 (d, *J* = 9.4 Hz, 1H), 7.93 – 7.67 (m, 3H), 7.58 (s, 1H), 7.42 (d, *J* = 8.7 Hz, 1H), 4.87 (dd, *J* = 26.4, 2.4 Hz, 4H), 3.63 (dt, *J* = 15.6, 2.4 Hz, 2H). **^{13}C NMR** (75 MHz, DMSO-d^6) δ 162.1, 159.3, 152.3, 148.6, 144.3, 142.2, 131.7, 131.3, 130.9, 129.9, 128.4, 124.8, 124.1, 115.3, 103.8, 100.1, 78.6, 78.5, 77.9, 53.6, 52.5.

Figure S9. ^{1}H and ^{13}C NMR characterization of prop-2-yn-1-*yl*-(*E*)-(5-(((prop-2-yn-1-yloxy)carbonyl)imino)-5*H*- benzo[a]phenoxazin-9-*yl*)carbamate (**3**) in DMSO.

UHPLC-MS analysis. The compounds underwent analysis through analytical UHPLC-MS employing an *Agilent 1200* series LC/MS system with a *Phenomenex SB C18* (1.8 µm, 2.1 × 50 mm) analytical column. The standard conditions for analytical UHPLC involved a linear gradient from 5 to 95% of solvent B over 15 minutes at a flow rate of 0.35 mL/min (Solvent A: water with 0.1% TFA, Solvent B: acetonitrile with 0.1% TFA). Detection of compounds occurred through UV absorption at 222, 270, and 330 nm. Electrospray Ionization Mass Spectrometry (ESI/MS) was conducted using an *Agilent 6120 Quadrupole LC/MS* model in positive scan mode, with direct injection of the purified compound solution into the MS detector.

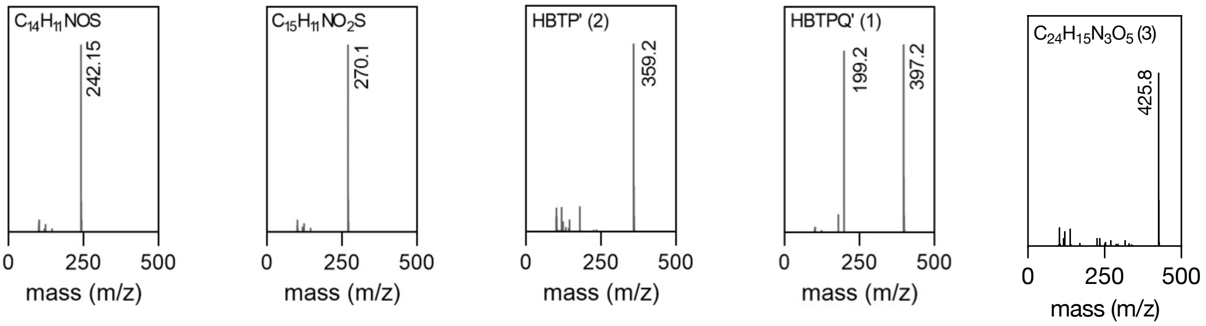

Figure S10. ESI-MS spectra of the compound synthesized in this route from left to right 2-(benzo[d]thiazol-2-yl)-4-methylphenol, 3-(benzo[d]thiazol-2-yl)-2-hydroxy-5-methylbenzaldehyde, (E)-4-(3-(benzo[d]thiazol-2-yl)-2-hydroxy-5-methylstyryl)-1-methylpyridin-1-ium (**2**), (E)-4-(3-(benzo[d]thiazol-2-yl)-5-methyl-2-(prop-2-yn-1-yloxy)styryl)-1-methylpyridin-1-ium, (**1**), and prop-2-yn-1-*yl* (*E*)-(5-(((prop-2-yn--yloxy) carbonyl)imino)-5H-benzo[a]phenoxazin-9-yl)carbamate (**3**), showing in each case peaks matching the expected mass.

Circular Dichroisms

Circular Dichroism experiments were made with a *Jasco-715* coupled with a *thermostat Nestlab RTE-111*. The settings used were the following: Acquisition range: 300-195nm; band width: 2.0 nm; resolution: 0.2 nm; accumulation: 5 scans; sensitivity 10 mdeg; response time: 0.25 s, speed: 100 nm/min. Measurements were made in a 2 mm cell at 25 °C. Samples contained 10 mM phosphate buffer pH 7.5 and 100 mM of NaCl, 5 µM peptide and 1 equiv. of [$PdCl_2$(COD)] (when present).

The mixtures were allowed to stand for 5 min before registering the spectra. The final spectra are the average of 5 scans and were processed using the "*smooth*" macro implemented in the program *Kaleidagraph* (v 3.5 by Synergy Software).

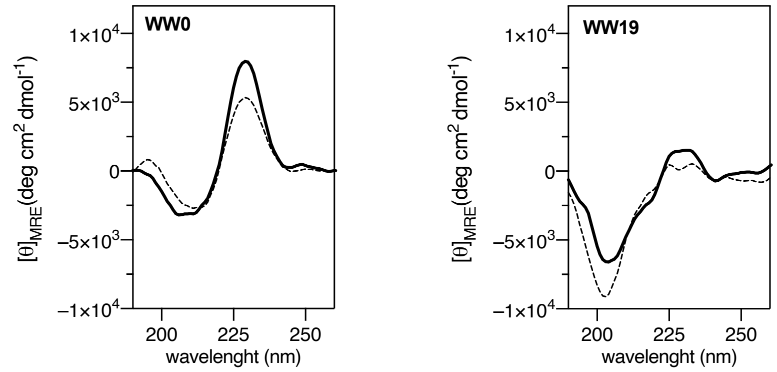

Figure S11. Circular dichroism spectra of 5 µM solutions of the non-coordinating WW peptides, **WW0** and the mono histidine **WW19**, before (thick solid lines), and after incubation with 1 equiv. of [$PdCl_2$(COD)] (dashed lines). Measurements were made in a 2 mm cell at 25 °C. Samples contained 10 mM phosphate buffer pH 7.5 and 100 mM of NaCl.

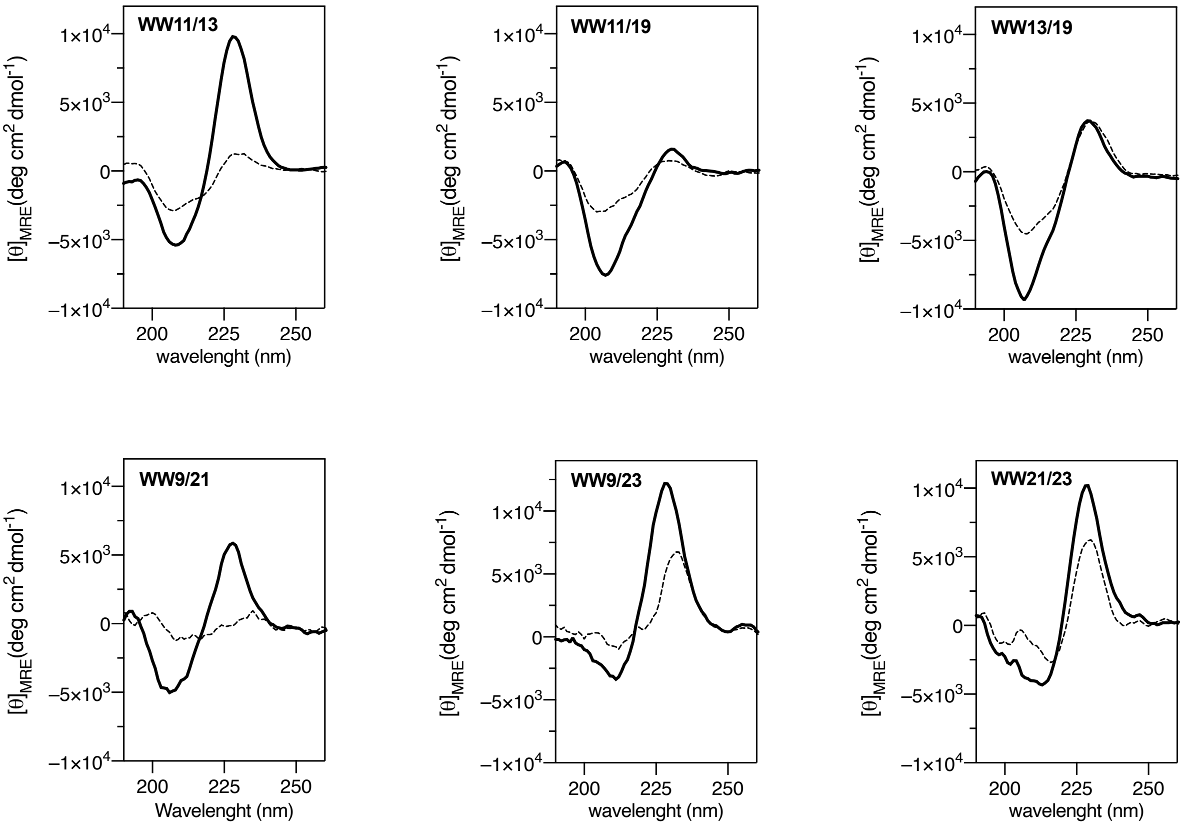

Figure S12. Circular dichroism spectra of 5 μM solutions of the WW peptides, before (thick solid lines), and after incubation with 1 equiv. of [$PdCl_2$(COD)] (dashed lines) and formation of their corresponding coordination complexes. Measurements were made in a 2 mm cell at 25 °C. Samples contained 10 mM phosphate buffer pH 7.5 and 100 mM of NaCl.

NMR of the WW peptide and its Pd complex

NMR experiments were recorded on a *Bruker Avance III* 600-MHz spectrometer (IRB Barcelona) equipped with a quadruple (^{1}H, ^{13}C, ^{15}N, ^{31}P) resonance cryogenic probe head and a z-pulse field gradient unit at 298 K using a 1 mM solution of each peptide—in the presence or absence of [$PdCl_2$(COD)]—1D proton spectra were recorded with a sweep width of 12000 Hz and 32 k data points. A total of 16 scans were accumulated with an acquisition time of 2.05 s. A Watergate w5 composite pulse was used to suppress the water signal.

^{1}H 2D-TOCSY and NOESY experiments were acquired in 90% H_2O/10% D_2O and used to assign the spin systems corresponding to the peptide resonances (*5–7*). For the 2D-NOESY experiments, mixing times of 300, 150, and 80 ms were acquired to minimize the impact of spin-diffusion in the assignments. Spin-locking fields of 8 kHz and 50 ms mixing time was used for the 2D-TOCSY experiments. All 2D spectral widths were 8000 Hz. The data size was 512 points in F1, indirect dimension, and 2048 points in F2, direct dimension. For each F1 value, 48 transients were accumulated in the NOESY and 32 in the TOCSY experiments respectively. Data were processed with a combination of exponential and shifted sine-bell window functions for each dimension followed by automated baseline and phase correction using *TopSpin 3.5* (© Bruker 2020). The 512×2 k data matrices were zero-filled to 2 k × 2 k (NOESY and TOCSY).

Structure Calculation. To prove that the primary structure (composition and connectivity) corresponds to the theoretical sequence, we have followed the sequence assignment strategy (*7*). We identified the characteristic spin system of every residue in the sequence, using the 2D-TOCSY experiment, and every spin system was connected to the following one via NOEs observed from the side chain of a given residue (*i*) to the amide proton of the following residue (*i*+1) as well as from the amide proton of (*i*) to the amide of (*i*+1). The full spin analysis as well as the assignment of the NOEs were carried out manually using CARA software (*8*). Distance restraints derived from the NOESY experiments were used for the NMR-based model building of the peptide in solution using unambiguously assigned peaks exclusively and the program CNS 1.2 (Crystallography and NMR system) (*9*). The protocol consisted of an implicit water simulated-annealing of 120

structures using 8,000 cooling steps followed by an explicit water refinement of the calculated structures using all experimental restraints during 1200 steps. The Pd coordination was not explicitly included in the calculation. To display the metal coordination, we manually optimized the His rotamers to facilitate the coordination and the Pd was added to the final model.

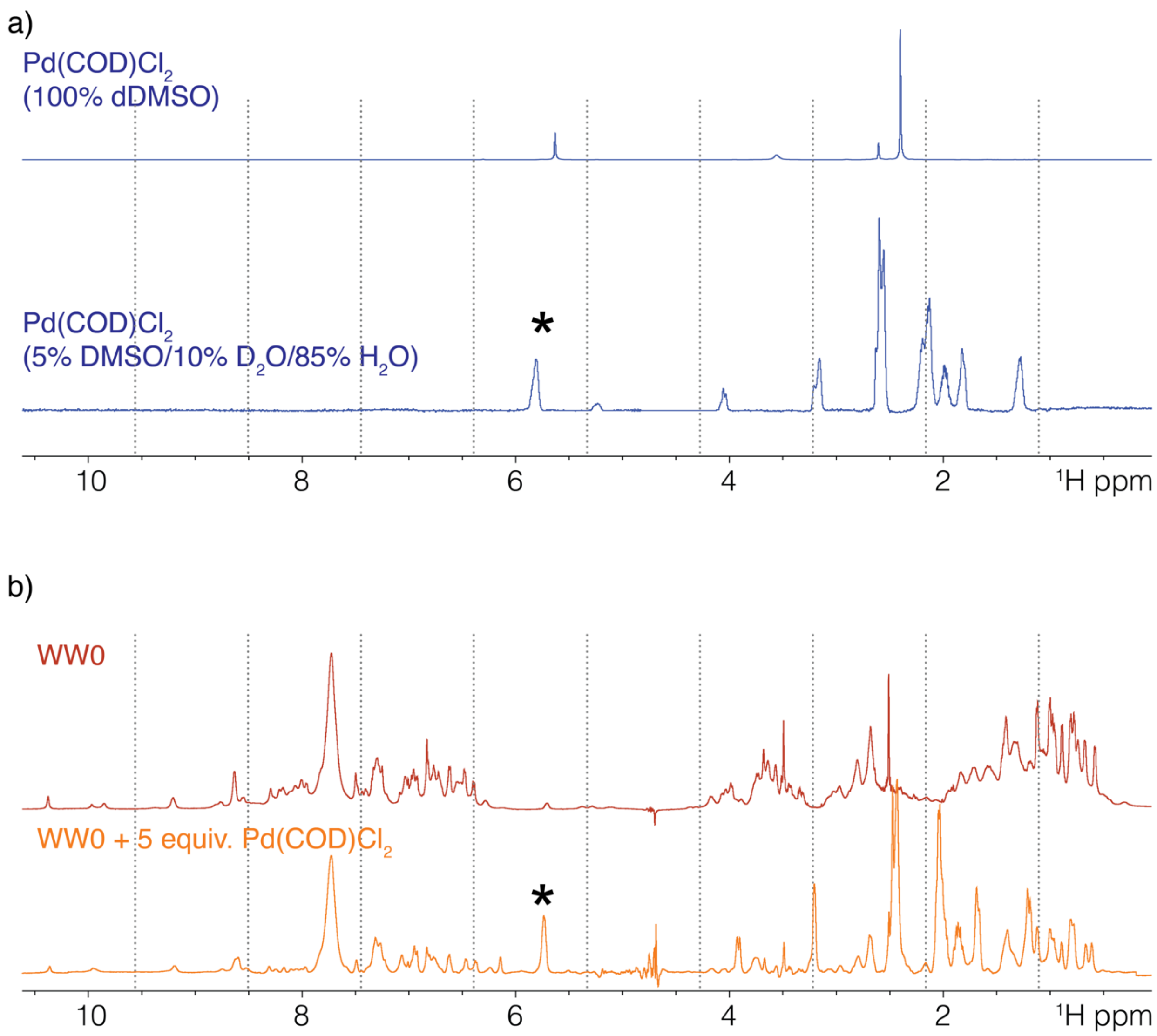

Figure S13. a) [$PdCl_2$(COD)]. 1D NMR spectra in 100% dDMSO and 5% dDMSO/10% D_2O/85% H_2O. b) **WW0** 1D NMR spectrum in the absence (top) and presence (bottom) of 5 equivalents of [$PdCl_2$(COD)]. Asterisk shows a signal corresponding to the unbound [$PdCl_2$(COD)].

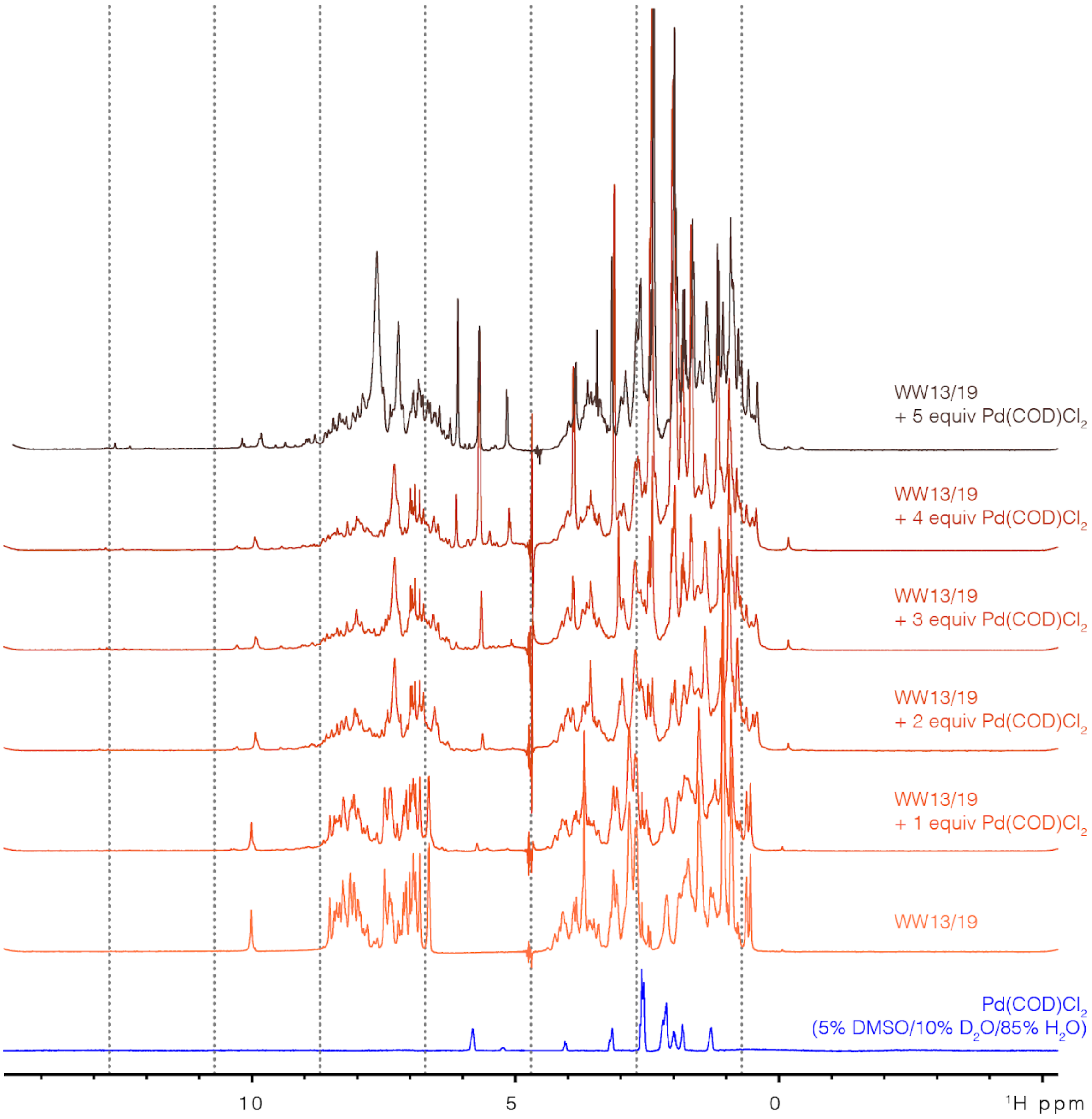

Figure S14. Titration of **WW13/19** with up to five equivalents of [$PdCl_2$(COD)]. Progressive folding of the peptide can be followed by Trp Hε peaks at ~10 ppm and general dispersion of the amide region (~ 6-9ppm).

Computational studies

BioMetAll statistics. *BioMetAll* (*10*) is a metal-binding prediction tool based on protein and peptide preorganization, considering exclusively the structure and disposition of the backbone. For this reason, several slightly different structures were considered, to account for variability across WW domain scaffolds. The 12 structures selected were: 1) NMR derived structures with PDB codes 1E0M, 1E0N, 1E0L (*1*), and 1ZR7 (*11*); 2) two snapshots of the most representative clusters along a 50 ns cMD for PDB codes 1E0M, 1E0N and 1ZR7; 3) two snapshots of the most representative clusters along a 200 ns cMD of the **WW0** prototype.

BioMetAll was instructed to search for mutations of any of the residues to a His-His motif, avoiding clashes with the backbone and sidechains at a distance shorter than 2 Å. Then, results were analyzed accounting for all mutation combinations with several probes that represent more than 40% of the total probes, to ensure that only relevant combinations are considered.

The most repeated mutations were in positions 11, 13, 17, 18-20 and 21, suggesting the β1-β2 turn as focal point for the metal binding site.

Classical Molecular Dynamics run for each structure to obtain different snapshots were setup with *xleap*, solvating the peptides with TIP3P water molecules in a box with a distance to the peptide of 10 Å. The system was neutralized with chloride ions. The atoms in the amino acids were represented with the AMBER14SB force field (*12*), while using GAFF force field for the remaining atoms. For the simulations, the OpenMM engine was used, following the OMM Protocol. It starts with an energy minimization of the whole system for 2000 steps, then the water molecules and side chains were heated up from 100 K to 300 K. Finally, MD under periodic boundary conditions were run for 50 ns in the case of the crystallographic sequences, and for 200 ns for the **WW0** sequence. Convergence of the trajectory was analyzed with *Ptraj* module from AmberTools18.

Pd coordination study. The energy calculation of the different coordination modes was carried out at the DFT level of theory using the program Gaussian16 using the hybrid functional B3LYP with the D3 version of Grimme's dispersion correction; the basis set used for the C, H, and N atoms was the 6-31+G(d,p), while for the Pd atom the Stuttgart/Dresden pseudopotential (SDD) was used (*13*). The convergence of forces and step size were considered converged at a tight level (1×10^{-5}) and the solvent was represented with the solvent-polarizable dielectric continuum model (SMD) (*14*). The structures of the models were optimized without any restrictions. The free energy (G) values are calculated incorporating the zero-point, thermal and entropy corrections to the potential energy value obtained directly from the SCF calculation. These are the values considered for comparison between the coordination modes.

Model	ΔG (kcal/mol)
εδ	0
δε	0.764
εε	1.760
δδ	-

It must be noted that for the Nδ/Nδ coordination mode the calculation showed a proton transfer indicating that both His residues protonated at the ε-N are not stable coordinated to Pd, so it was impossible to calculate the energy.

Gaussian Accelerated Molecular Dynamics study. The metal coordinating parameters were derived from the DFT calculations through the Seminario method through MCPB.py tool (*15*), and the charges were obtained with the restrained electrostatic potential (RESP) model (*16*). The systems with and without Pd were first submitted to a cMD for stabilization and then a total of 26 000 000 steps of different equilibrations. Finally, 400 ns of production of the accelerated molecular dynamics are run, enlarged in the cases where it didn't converge up to 500ns. To assess the conformational properties of the triple-stranded β-sheet, Ramachandran plots were devised for each peptide using the density estimates from the RamachanDraw program (available at https://github.com/alxdrcirilo/RamachanDraw), MDtraj (*17*), and Numpy (*18*) to process the data and Matplotlib to design and execute the plots (*19*).

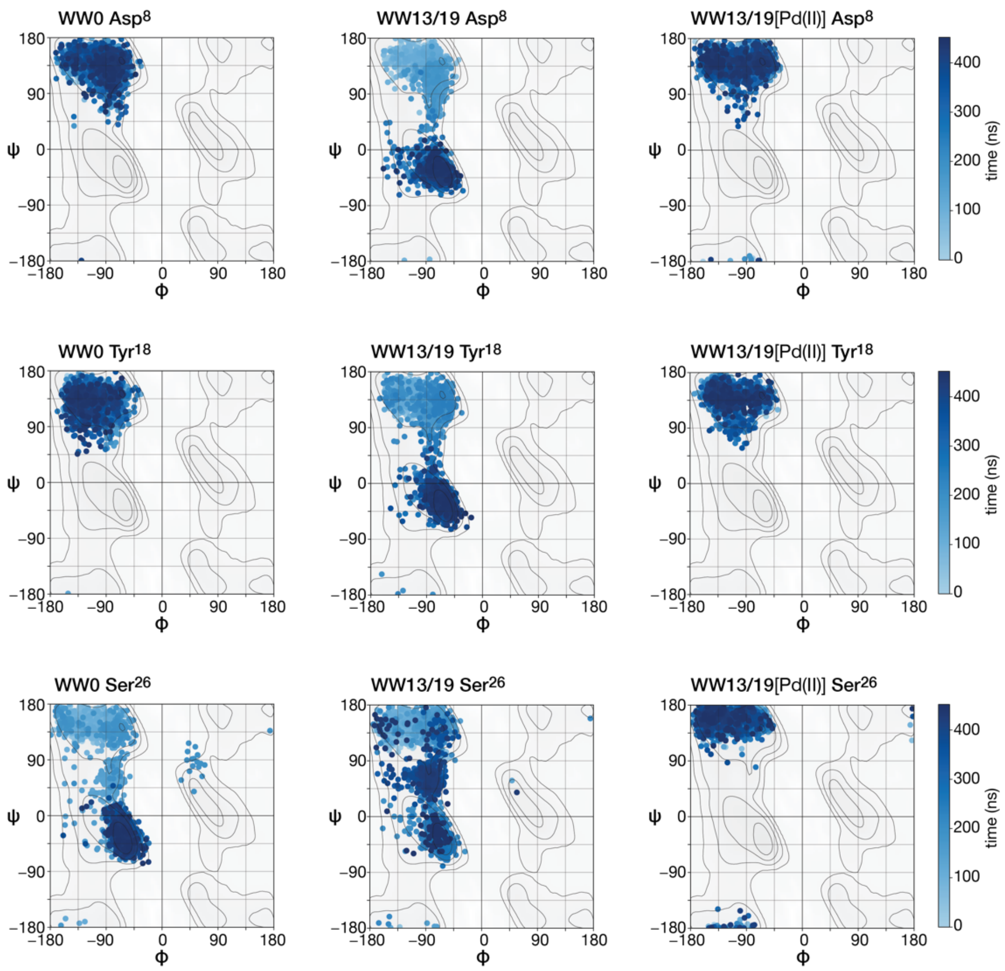

Figure S15. Ramachandran plots of representative residues in the three β-strands (Asp[8] in β1, Tyr[18] in β2, and Ser[26] in β3) along the GaMD simulations of **WW0**, **WW13/19** and **WW13/19**[Pd(II)]. In **WW0** (first column) Asp[8] and Tyr[18] are stable in the β region of the Ramachandran plot, but Ser[26] shows higher conformational freedom, typically observed in WW domains. **WW13/19** (second column) is poorly folded, as can be seen by the exploration of other regions of the Ramachandran plot by the three residues. Ser[26] is also the one showing higher heterogeneity. **WW13/19**[Pd(II)] (third column) shows Ramachandran plots resembling those of the reference **WW0**, with Asp[8] and Tyr[18] concentrated in the β region of the plot. Notably, Ser[26] is now also restricted to the β region of the plot in the first part of the simulation (up to 400 ns).

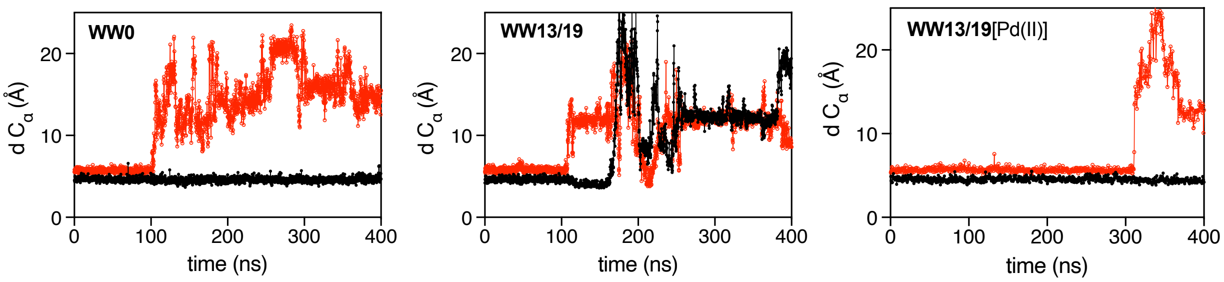

Figure S16. Evolution of the distance between β strands along the GaMD trajectories for **WW0**, **WW13/19** and **WW13/19**[Pd(II)]. Distances are measured between the Cα of the central residues of each strand: Glu[8]-Tyr[18] (for distance between strands β1 and β2, in black) and Tyr[18]-Ser[26] (for distance between stands β2 and β3, shown in red). **WW13/19** shows higher flexibility and the strands fall apart rapidly, but **WW13/19**[Pd(II)] shows a highly stable β1-β2 hairpin and β3 remains stable and attached to the β-sheet for the most part of the simulation.

In vitro catalytic studies

The catalytic deprotection of **probe 1** to release the uncaged product **2** was performed in a 2.0 mL HPLC-vial with screw cap. For this purpose, a fresh solution of **1** (10 μL, 20 mM in DMSO, 1.0 equiv.) was added to PBS (990 μL), and to the resulting mixture was added a solution of the [$PdCl_2$(COD)] (1 μL, 20 mM in DMSO, 0.1 equiv.). The reaction mixture was kept for 24 h at 37 °C under stirring at 1000 rpm. After that time, 50 μL of the reaction was taken and diluted to 100 μL with MeOH and analyzed by reverse-phase HPLC-MS. The results were treated according to the calibration curve, in which coumarin was used as internal standard. Every value is the average value of two independent measurements.

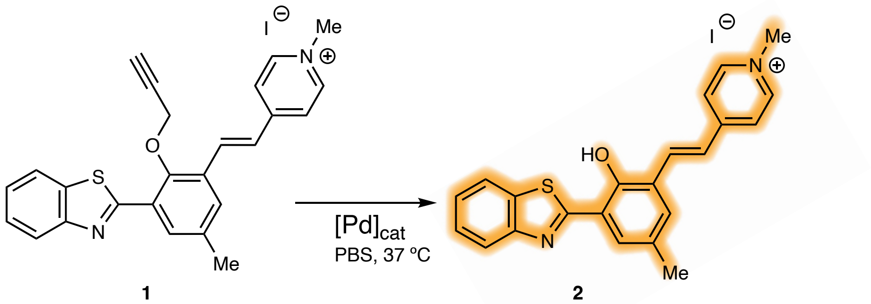

Scheme S3. Catalytic depropargylation reaction of probe **1**.

The experiments using an ultra-diafiltration with Amicon® Ultra 0.5 mL Centrifugal Filters (3k device) to remove the remaining free [$PdCl_2$(COD)], were carried out as described above but before the reactions and after the coordination, we filtrated the crude of the peptides and the palladium salt with the filters (x3 times), using the same buffer (PBS).

Depropargylation kinetics of the HBTPQ' probe 1

A 200 μM solution of probe **1** in 10 mM phosphate buffer, 100 mM NaCl, pH = 7.5, was incubated for 15h with 20 μM **WW0** or **WW13/19** and [$PdCl_2$(COD)] in a 1:1 ratio for 1h, then the metallopeptides were separated from the uncomplexed [$PdCl_2$(COD)] by ultra-diafiltration with 3 kDa Amicon centrifugal filters. Catalysis was carried out at 37 °C measuring the fluorescence at 620 nm upon excitation at 405 nm every 15 min in PBS (plate reader).

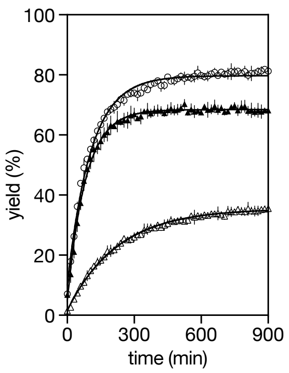

Figure S17. Kinetic traces of HBTPQ' depropargylation. Probe **1** in in 10 mM phosphate buffer, 100 mM NaCl, pH = 7.5 in the presence of **WW13/19** + [$PdCl_2$(COD)] (○); **WW0** + [$PdCl_2$(COD)] (△); [$PdCl_2$(COD)] (▲). Lines show the best fit to a pseudo-first kinetic model of the depropargylation profiles (%yield $Y = Y_0 + (Y_{max} - Y_0) \times (1 - e^{-kt})$, as described in Domingos *et al.* (*20*).

General information for the experiments with cell cultures

All steps were performed on a sterile clean bench *Tesltar AV-100* at room temperature. Solutions stored in a fridge were warmed beforehand in a water bath (37 °C). Unless otherwise specified, all incubations were performed in FBS-DMEM (DMEM containing 5% of fetal bovine serum).

Cell Culture: All cell lines were cultured in DMEM (Dulbecco´s modified Eagle´s medium), 5 mM glutamine, penicillin (100 units/mL) and streptomycin (100 units/mL) (all from Invitrogen). Proliferating cultures were maintained in a 5% CO_2 humidified incubator at 37 °C. For all the experiments, cells were seeded in the corresponding well at the indicated concentration two days before treatment.

Fluorescence microscopy: All images were obtained with an *Andor Zyla* mounted on a *Nikon TiE*. Confocal images were acquired in an *Andor Dragonfly High Speed Confocal Platform*. Images were further processed with *Image J* or *NIS software* (*Nikon*). Microscopy settings: The filter sets for the observation of the fluorescence of the products were as follows: Probe **2**: Widefield: LED λ excitation: 385 nm. Filter cube: BP 375/28x nm, LP 515lp nm and DM 415 nm; TMR and Probe **4**: Widefield: LED λ excitation: 550 nm. Filter cube TRITC-B-000 (Semrock): BP 543/22 nm, LP 593/40 nm and DM 562 nm. Confocal: Laser excitation: 561 nm. LP 620/60 and DM 567 nm.

Plate reader: A *Tecan Infinite F200Pro* microplate reader was used to measure directly in *Costar* cell culture 96-well plates UV-Vis absorbance for the MTT viability assays.

Cell internalization studies. Cells were seeded on glass-bottom plates 48 h before treatment. Before the addition to cells, peptides were pre-incubated with [$PdCl_2$(COD)] (1:1) in water for 1 h, to ensure the formation of the palladium complexes Culture medium was then removed and DMEM containing 5% fetal bovine serum (FBS-DMEM) and peptides (5 µM) or palladopeptides (5 µM) were added. Before the addition to cells, peptides were pre-incubated with [$PdCl_2$(COD)] (1:1) in water for 10 min, to ensure the formation of the palladium complexes. After 30 min, cells were washed twice with PBS and the medium replaced with fresh FBS-DMEM and observed under the microscope with appropriate filters. Digital pictures of the different samples were taken under identical conditions of gain and exposure.

CTFC measurements. We have processed the different images of internalization experiments with *Image J* program using the analyze tool, obtaining a mean value of corrected total cell fluorescence (CTFC) regarding TMR fluorophore.

Inhibitors assays. Cytometry: Flow cytometry was performed on a *Guava easyCyteTM* cytometer. Data analysis was performed with *InCyte* software included in *GuavaSoft 3.2* (*Millipore*).

To know more about the internalization process, we performed experiments with a series of endocytosis inhibitors. For that, we incubated Hela Cells with each of the inhibitors for 30 min and, after washing, we added the preformed metallopeptide **TMR-WW13/19**[Pd(II)]. We measured the emission of the TMR by cytometry and quantified the internalization in each case. Dynasore and EIPA were able to partially inhibit the uptake, suggesting that our palladopeptide uses an energy-dependent process to internalize in mammalian cells. Concretely the assay suggests the block of dynamin, a key protein for vesicle fission and the micropinocytosis process, were the most affected pathways.

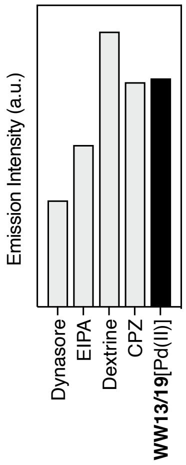

Figure S18. Endocytosis inhibitors. HeLa cells were incubated for 30 min with each inhibitor prior to the addition of the preformed TMR-**WW13/19**[Pd(II)] metallopeptide, and the emission was measured with cell cytometry.

Intracellular reactions. For intracellular reactions probe **1** and probe **3** were tested.

Cells were seeded on glass-bottom plates 48 h before treatment. Culture medium was removed and 300 µL of FBS-DMEM containing probe **1** (50 µM) were added. After 1 hour incubation, cells were washed twice with FBS-DMEM and 300 µL of a solution of the palladopeptides (50 µM) (peptides were preincubated with [$PdCl_2$(COD)] (1:1) in water for 1 h before the addition to cells) in FBS-DMEM were added. After a 1 h incubation, cells were washed twice with FBS-DMEM and replace with fresh FBS-DMEM to observe under the microscope with appropriate filters. Digital pictures of the different samples were taken under identical conditions of gain and exposure.

Most relevant images from *in cellulo* experiments:

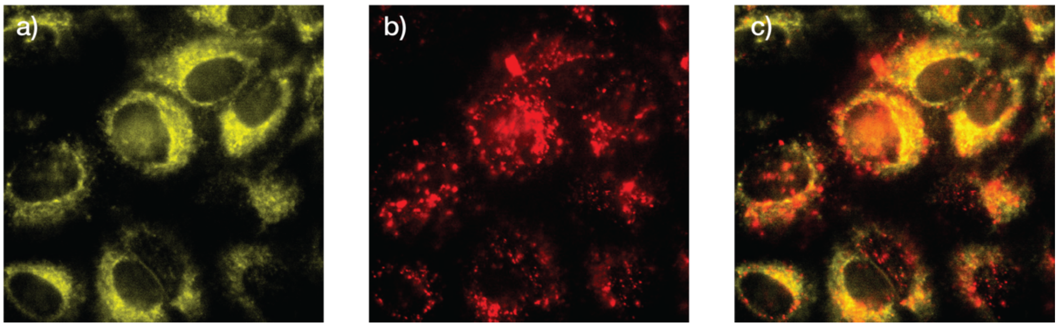

Figure S19. Cellular depropargylation of probe **1** with TMR-**WW13/19**[Pd(II)] in HeLa cells. Fluorescence microscopy of HeLa cells incubated with a 50 µM solution of the fluorogenic probe **1** for 1 h, washed twice with FBS-DMEM, and incubated for 1 h with 50 µM of a premade mixture of TMR-**WW13/19** with [$PdCl_2$(COD)], after which the cells are imaged. A) Emission of the deprotected probe **2**; b) Emission for the TMR fluorophore attached to **WW13/19**; c) Colocalization of both images.

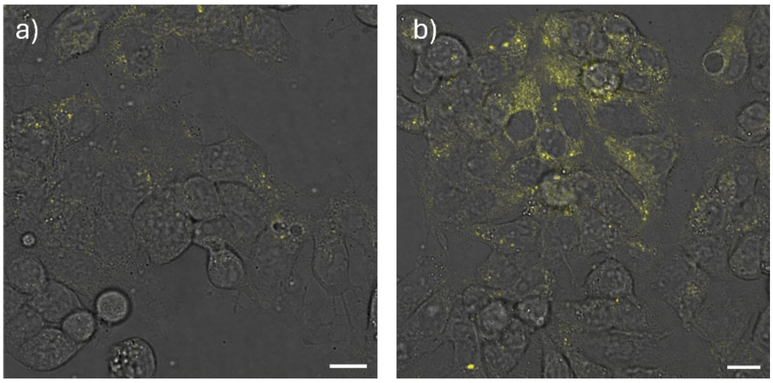

Figure S20. Cellular depropargylation of probe **1** with **WW13/19**[Pd(II)] in HepG2 cells. Fluorescence microscopy of HeLa cells incubated with a 50 µM solution of the fluorogenic probe **1** for 1 h, washed twice with FBS-DMEM, and incubated for 1 h with 50 µM of a premade mixture of **WW13/19** with [$PdCl_2$(COD)], after which the cells are imaged. a) Emission of probe **1**; b) Emission after the depropargylation reaction.

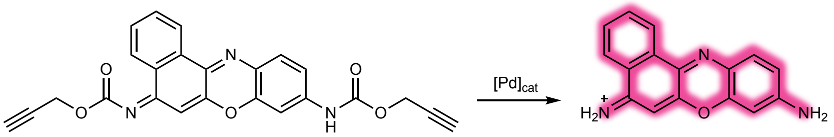

Scheme S4. Catalytic depropargylation reaction of the cresyl violet probe **3**.

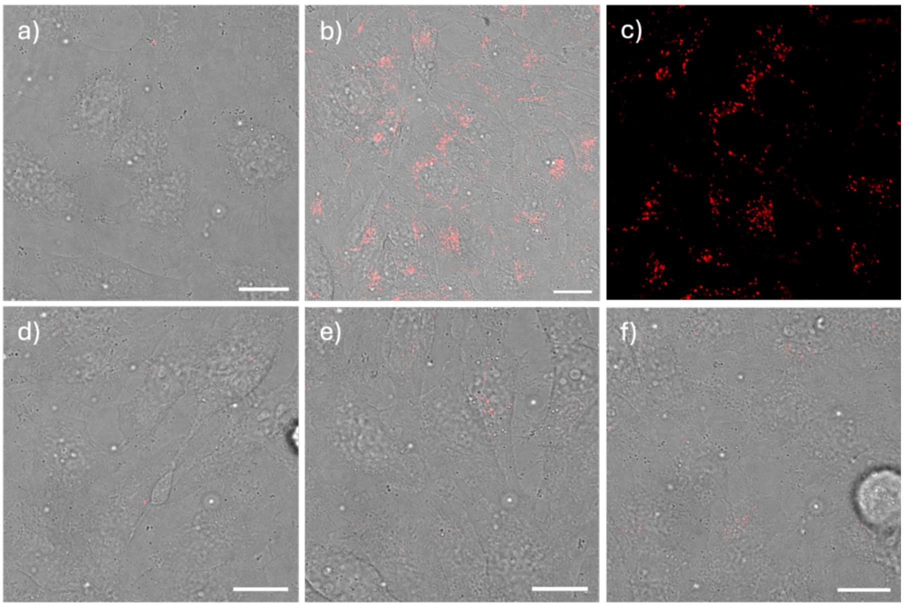

Figure S21. Depropargylation of probe **3** in HeLa cells. Fluorescence microscopy of HeLa cells incubated with: a) probe **3** (50 µM) for 1 h; probe **3** (50 µM) for 1 h, washed twice with FBS-DMEM and incubated for 6 h with 50 µM of: b and c) a premade palladopeptide **WW13/19**[Pd(II)]; d) **WW0**; e) **WW19**; f) [$PdCl_2$(COD)].

CTFC measurements. We have processed the different images of intracellular reactions experiments with Image J program using the analyze tool, obtaining a mean value of corrected total cell fluorescence (CTFC) regarding **2**.

ICP-MS. The metal content of the samples was determined by ICP-MS (*Agilent 7700x*) with a sample introduction system consisting of a *Micromist* glass low-flow nebulizer, a double-pass glass spray chamber with a Peltier system (3 °C) and a quartz torch. For the ICP measurements, 100.000 cells/mL were seeded in 6-well plated two days before treatment with metallopeptide resulting from **WW13/19** and [$PdCl_2$(COD)] or [$PdCl_2$(COD)], in FBS-DMEM for 1 h. Cells were then washed twice with PBS and lysed in 70% HNO_3. The obtained lysates were digested in duplicate with HNO_3/H_2O_2 by heating with microwave energy before being analyzed.

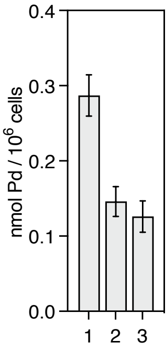

Figure S22. ICP-ms assay. ICP-MS analysis of cellular extracts obtained after exposing HeLa cells to the metalloenzyme or the controls, showed more amount of palladium when incubated with **WW13/19**[Pd(II)]

HPLC MS product detection. HeLa cells were seeded in 6-well plate 48 h before the experiment and incubated for 1 h with a 50 μM solution of the probe **1**. After washing twice with PBS, the cells were treated for 2 h with a premade mixture 1:1 (50 μM) mixture of the peptide **WW13/19** and [$PdCl_2$(COD)]. After washing twice with PBS, 1 mL of MeOH is added to each well and the extracted contents of the cells are collected after 5 min. These solutions are concentrated at a rotary evaporator and re-dissolved in MeCN for analysis by reverse-phase HPLC-MS.

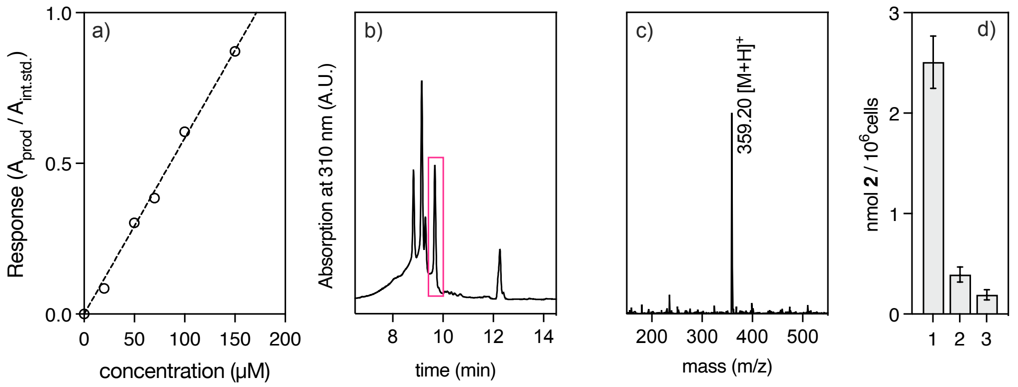

Figure S23. a) Calibration curve for product **2**; b) Reverse-phase HPLC-MS trace of the methanolic cell extracts of the intracellular reaction catalyzed with **WW13/19**[Pd(II)], indicating the peak corresponding to the deprotected product **2**; c) MS of the peak highlighted in b), corresponding to the mass of the depropargylated probe; d) Quantification of the internalized probe upon incubation with 1) **WW13/19**[Pd(II)]; 2) 50 μM **WW19** and [$PdCl_2$(COD)] (1:1); 3) 50 μM neat [$PdCl_2$(COD)].

Catalytic turnover number (TON)

Combining the measurements of the product amount by HPLC-MS(ESI) and palladium amount by ICP-MS, it is possible to calculate turnover numbers with a relatively good accuracy. Indeed, we found a turnover of 9 when incubated the metalloenzyme in second place and 5 if we incubated first with it. The results demonstrated the first TON calculated inside the cells using a protein scaffold and represent the first example of artificial catalytic mini-enzyme capable of working in the native living environments.

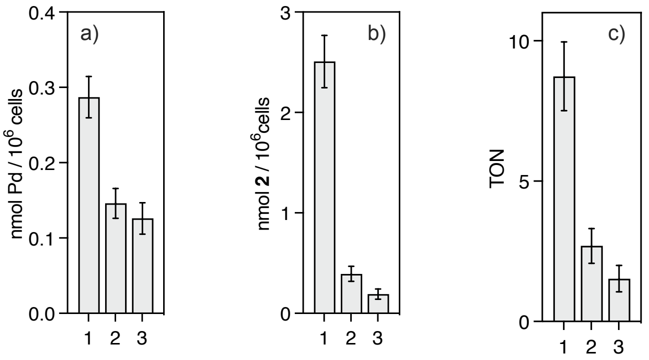

Figure S24. Calculation of TON. a) ICP-MS analysis of cellular extracts in Figure S22; b) Internalized probe as shown in Figure S23d; c) TON calculated as amount of product divided by the amount of catalyst. In all graphs**,** 1) 50 μM **WW13/19**[Pd(II)]; 2) 50 μM **WW19** and [$PdCl_2$(COD)] (1:1); 3) 50 μM neat [$PdCl_2$(COD)].

Viability test (MTT assay). The toxicity of **WW13/19**, the palladopeptide **WW13/19**[Pd(II)], or the precursor salt [$PdCl_2$(COD)] were tested by MTT assays in HeLa cells as follows (*21*): 100,000 cells per well were seeded in 96 well plates two days before treatment with different concentrations of the catalysts/peptides. After 24 h of incubation, HEPES containing 3-(4,5-dimethylthiazol-2-yl)-2,5-diphenyl tetrazolium bromide (MTT) was added to the cell culture medium to a final concentration of 0.5 mg/mL. Cells were then incubated for 4 h to allow the formation of formazan precipitates by metabolically active cells. A detergent solution of 10% SDS (sodium dodecyl sulphate) and 0.01 M HCl was then added, and the plate was incubated overnight at room temperature to allow the solubilization of the precipitates. The quantity of formazan in each well, which is directly proportional to the number of viable cells, was measured by recording changes in absorbance at 570 nm in a *Tecan Infinite F200 PRO* microtiter plate reading spectrophotometer.

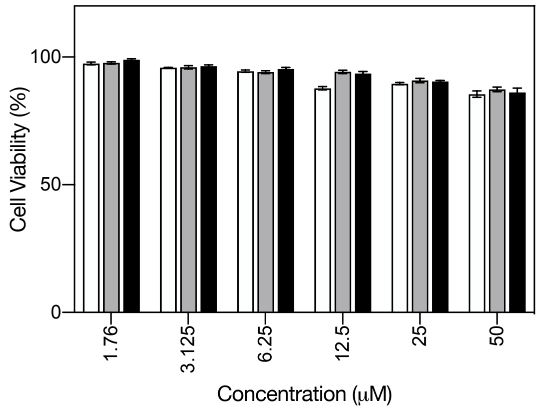

Figure S25. Toxicity assay. HeLa cells were incubated with **WW13/19** (white bars), the palladopeptide **WW13/19**[Pd(II)], or the precursor salt [$PdCl_2$(COD)] at different concentrations, for 24 h at 37 °C. Finally, the cells were washed two times with PBS before carrying out the MTT assays. Data are represented as them mean +/- SEM for experimental repeated in three independent times. The palladopeptide **WW13/19**[Pd(II)] was prepared by mixing the peptide **WW13/19** with [$PdCl_2$(COD)] (1:1 ratio) in water for 10 min before the addition to the cells.

References

1. M. J. Macias, V. Gervais, C. Civera, H. Oschkinat, Structural analysis of WW domains and design of a WW prototype. *Nat. Struct. Biol.* **7**, 375–379 (2000).
2. S. Learte-Aymamí, C. Vidal, A. Gutiérrez-González, J. L. Mascareñas, Intracellular Reactions Promoted by Bis(histidine) Miniproteins Stapled Using Palladium(II) Complexes. *Angew. Chem. Int. Ed Engl.* **59**, 9149–9154 (2020).
3. T. Gao, P. Xu, M. Liu, A. Bi, P. Hu, B. Ye, W. Wang, W. Zeng, A water-soluble ESIPT fluorescent probe with high quantum yield and red emission for ratiometric detection of inorganic and organic palladium. *Chem. Asian J.* **10**, 1142–1145 (2015).
4. J. Clavadetscher, E. Indrigo, S. V. Chankeshwara, A. Lilienkampf, M. Bradley, In-Cell Dual Drug Synthesis by Cancer-Targeting Palladium Catalysts. *Angew. Chem. Int. Ed Engl.* **56**, 6864–6868 (2017).
5. A. Bax, D. G. Davis, Practical aspects of two-dimensional transverse NOE spectroscopy. *J. Magn. Reson.* **63**, 207–213 (1985).
6. S. Macura, R. R. Ernst, Elucidation of cross relaxation in liquids by two-dimensional N.M.R. spectroscopy. *Mol. Phys.* **41**, 95–117 (1980).
7. K. Wüthrich, *NMR of Proteins and Nucleic Acids* (Wiley, Nashville, TN, 1986)*Baker Lecture Series*.
8. X. Ramirez-Espain, L. Ruiz, P. Martin-Malpartida, H. Oschkinat, M. J. Macias, Structural characterization of a new binding motif and a novel binding mode in group 2 WW domains. *J. Mol. Biol.* **373**, 1255–1268 (2007).
9. A. T. Brunger, Version 1.2 of the Crystallography and NMR system. *Nat. Protoc.* **2**, 2728–2733 (2007).
10. J.-E. Sánchez-Aparicio, L. Tiessler-Sala, L. Velasco-Carneros, L. Roldán-Martín, G. Sciortino, J.-D. Maréchal, BioMetAll: Identifying Metal-Binding Sites in Proteins from Backbone Preorganization. *J. Chem. Inf. Model.* **61**, 311–323 (2021).
11. Y. Kato, Y. Hino, K. Nagata, M. Tanokura, Solution structure and binding specificity of FBP11/HYPA WW domain as Group-II/III. *Proteins* **63**, 227–234 (2006).
12. V. Hornak, R. Abel, A. Okur, B. Strockbine, A. Roitberg, C. Simmerling, Comparison of multiple Amber force fields and development of improved protein backbone parameters. *Proteins* **65**, 712–725 (2006).
13. A. W. Ehlers, M. Böhme, S. Dapprich, A. Gobbi, A. Höllwarth, V. Jonas, K. F. Köhler, R. Stegmann, A. Veldkamp, G. Frenking, A set of f-polarization functions for pseudo-potential basis sets of the transition metals Sc-Cu, Y-Ag and La-Au. *Chem. Phys. Lett.* **208**, 111–114 (1993).
14. A. V. Marenich, C. J. Cramer, D. G. Truhlar, Universal solvation model based on solute electron density and on a continuum model of the solvent defined by the bulk dielectric constant and atomic surface tensions. *J. Phys. Chem. B* **113**, 6378–6396 (2009).
15. P. Li, K. M. Merz Jr, MCPB.py: A Python Based Metal Center Parameter Builder. *J. Chem. Inf. Model.* **56**, 599–604 (2016).
16. C. I. Bayly, P. Cieplak, W. Cornell, P. A. Kollman, A well-behaved electrostatic potential based method using charge restraints for deriving atomic charges: the RESP model. *J. Phys. Chem.* **97**, 10269–10280 (1993).
17. R. T. McGibbon, K. A. Beauchamp, M. P. Harrigan, C. Klein, J. M. Swails, C. X. Hernández, C. R. Schwantes, L.-P. Wang, T. J. Lane, V. S. Pande, MDTraj: A Modern Open Library for the Analysis of Molecular Dynamics Trajectories. *Biophys. J.* **109**, 1528–1532 (2015).
18. C. R. Harris, K. J. Millman, S. J. van der Walt, R. Gommers, P. Virtanen, D. Cournapeau, E. Wieser, J. Taylor, S. Berg, N. J. Smith, R. Kern, M. Picus, S. Hoyer, M. H. van Kerkwijk, M. Brett, A. Haldane, J. F. Del Río, M. Wiebe, P. Peterson, P. Gérard-Marchant, K. Sheppard, T. Reddy, W. Weckesser, H. Abbasi, C. Gohlke, T. E. Oliphant, Array programming with NumPy. *Nature* **585**, 357–362 (2020).
19. J. D. Hunter, Matplotlib: A 2D Graphics Environment. *Comput. Sci. Eng.* **9**, 90–95 (May-June 2007).
20. G. M. Dal Forno, E. Latocheski, C. D. Navo, B. L. Albuquerque, A. L. St John, F. Avenier, G. Jiménez-Osés, J. B. Domingos, Interplay of chloride levels and palladium(II)-catalyzed O-deallenylation bioorthogonal uncaging reactions. *Chem. Sci.* **15**, 4458–4465 (2024).
21. J. van Meerloo, G. J. L. Kaspers, J. Cloos, Cell sensitivity assays: the MTT assay. *Methods Mol. Biol.* **731**, 237–245 (2011).
